# Copy number variations in the genome of the Qatari population

**DOI:** 10.1186/s12864-015-1991-5

**Published:** 2015-10-22

**Authors:** Khalid A. Fakhro, Noha A. Yousri, Juan L. Rodriguez-Flores, Amal Robay, Michelle R. Staudt, Francisco Agosto-Perez, Jacqueline Salit, Joel A. Malek, Karsten Suhre, Amin Jayyousi, Mahmoud Zirie, Dora Stadler, Jason G. Mezey, Ronald G. Crystal

**Affiliations:** Department of Genetic Medicine, Weill Cornell Medical College in Qatar, Doha, Qatar; Division of Translational Medicine, Sidra Medical Research Centre, Doha, Qatar; Department of Physiology and Biophysics, Weill Cornell Medical College in Qatar, Doha, Qatar; Computer and Systems Engineering, Alexandria University, Alexandria, Egypt; Department of Genetic Medicine, Weill Cornell Medical College, 1300 York Avenue, Box 164, New York, NY 10065 USA; Department of Medicine, Hamad Medical Corporation, Doha, Qatar; Department of Medicine, Weill Cornell Medical College in Qatar, Doha, Qatar; Department Biological Statistics and Computational Biology, Cornell University, Ithaca, NY USA

**Keywords:** Copy number variation, Next-generation sequencing, Genotyping, Genomics, Mendelian disease, Qatar

## Abstract

**Background:**

The populations of the Arabian Peninsula remain the least represented in public genetic databases, both in terms of single nucleotide variants and of larger genomic mutations. We present the first high-resolution copy number variation (CNV) map for a Gulf Arab population, using a hybrid approach that integrates array genotyping intensity data and next-generation sequencing reads to call CNVs in the Qatari population.

**Methods:**

CNVs were detected in 97 unrelated Qatari individuals by running two calling algorithms on each of two primary datasets: high-resolution genotyping (Illumina Omni 2.5M) and high depth whole-genome sequencing (Illumina PE 100bp). The four call-sets were integrated to identify high confidence CNV regions, which were subsequently annotated for putative functional effect and compared to public databases of CNVs in other populations. The availability of genome sequence was leveraged to identify tagging SNPs in high LD with common deletions in this population, enabling their imputation from genotyping experiments in the future.

**Results:**

Genotyping intensities and genome sequencing data from 97 Qataris were analyzed with four different algorithms and integrated to discover 16,660 high confidence CNV regions (CNVRs) in the total population, affecting ~28 Mb in the median Qatari genome. Up to 40 % of all CNVs affected genes, including novel CNVs affecting Mendelian disease genes, segregating at different frequencies in the 3 major Qatari subpopulations, including those with Bedouin, Persian/South Asian, and African ancestry. Consistent with high consanguinity levels in the Bedouin subpopulation, we found an increased burden for homozygous deletions in this group. In comparison to known CNVs in the comprehensive Database of Genomic Variants, we found that 5 % of all CNVRs in Qataris were completely novel, with an enrichment of CNVs affecting several known chromosomal disorder loci and genes known to regulate sugar metabolism and type 2 diabetes in the Qatari cohort. Finally, we leveraged the availability of genome sequence to find suitable tagging SNPs for common deletions in this population.

**Conclusion:**

We combine four independently generated datasets from 97 individuals to study CNVs for the first time at high-resolution in a Gulf Arab population.

**Electronic supplementary material:**

The online version of this article (doi:10.1186/s12864-015-1991-5) contains supplementary material, which is available to authorized users.

## Background

The Qatari peninsula, located on the eastern coast of the Arabian Peninsula, is at a major crossroads of human migration [1]. This geographical location has led to several waves of settlement over the past millennia, creating unique ethnic ancestries that form the present day’s population. We previously described 3 major genetic subgroups of Qataris, including those of Bedouin (Q1), Persian-South Asian (Q2), and African ancestry (Q3) [2–4]. Although all three share a common environment, there is a significant level of segregation among the populations, with a higher level of consanguinity observed in Q1 and to a certain extent Q2, over Q3, leading to the formation of 3 distinguishable genetic pools. Exome sequencing has shown that each subpopulation has different predispositions to various Mendelian diseases, an observation with consequences for public health planning in the context of pre-existing premarital screening programs [5].

Whereas classic Mendelian disorders are generally defined by single nucleotide polymorphism’s (SNPs), there is increasing evidence that copy number variation (CNV) also play a significant role in disease [6–9]. CNVs in the 0.5 Kb to several Mb size range account for up to 4 % of the human genome, and generally behave like SNPs – having a detectable minor allele frequency, existing in linkage disequilibrium (LD) with neighboring SNPs on variable length haplotypes and being inherited in Mendelian fashion [7, 10]. Unlike SNPs, however, CNVs could be extremely multi-allelic (>3 segregating alleles), and may therefore contribute to continuous traits by causing a spectrum of variation in gene dosage [11]. Though common CNVs have traditionally shown relatively poor association with common disease [12], intermediate frequency (1-10 %) and rare (<1 %) but recurrent CNVs cause a variety of disorders, including extreme obesity, congenital heart disease, and a wide spectrum of neurological and developmental disorders [13–24].

Because of the high degree of consanguinity in the Qatari population, it is likely that CNVs, alongside SNPs and indels, play a role in the inherited disease risk burden in this population [5, 10, 25]. Further, because of the cultural segregation of the major genetic groups (Q1, Q2, and Q3) within the Qatari population, it is likely that there are novel CNV Regions (CNVRs) specific to each group, representing distinct subpopulation histories and risk for disease. In the context of these considerations, we have used complete genome sequencing along with SNP microarray analysis of 97 Qataris (57 Q1, 20 Q2, 20 Q3) to assess the spectrum of CNVs and CNVRs in the Qatari population, representing the first high-resolution, genome-wide assessment of the burden of small to medium-sized chromosomal deletions and duplications in a Middle Eastern Arab population. This work on the CNV class of mutations is complementary to a study from our group describing ancestry, polymorphisms and disease susceptibility from the single nucleotide variant class of mutations from these same individuals [26].

## Results

### Detection of CNVs

**Fig. 1:**
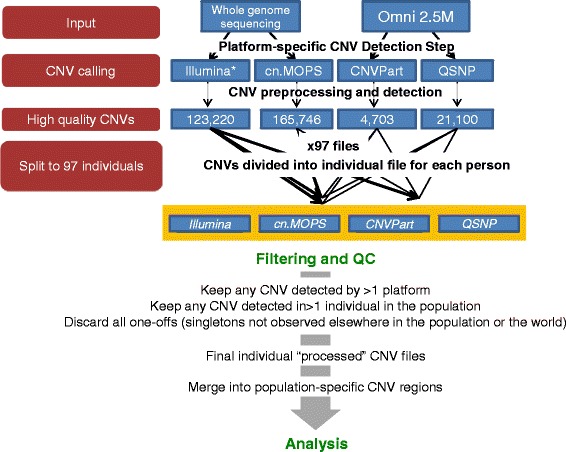
CNV analysis strategy. CNV detection in Qataris was assessed at two tiers. First, CNVs were called in 100 individuals using two algorithms each, on two primary input datasets: genotyping array (OMNI2.5 M) and next-generation whole genome sequencing reads (Illumina PE 100 bp, Mean Depth: 37X). A size cut-off of at least 5 consecutive probes for genotyping data and at least 5 consecutive windows for whole genome sequencing data was used to increase specificity (see Methods). Three samples with an unusually high number of CNVs were first removed from the population (see Additional file 1: Figure S1). In the second step, high-quality CNVs from the remaining 97 subjects called by all 4 platforms were distributed into 97 individual files. CNVs were first compared intra-individuals and retained if observed by more than one algorithm. If no overlap was detected within the individual, the CNV was compared inter-individuals to detect a second occurrence in the remaining 97 individuals. CNVs observed only once in the entire sample were discarded. CNVs passing these filters were merged across the population to generate population level CNV regions (CNVRs), which were taken into the detailed analysis steps. *Denotes data was provided as-is from proprietary Illumina Genome Network sequencing pipeline without the ability of the user to alter parameters

To identify CNVs in the Qatari population, primary data was obtained from arrays and whole-genome sequencing sources, and then called and integrated as described in methods (Fig. 1). Briefly, CNV calls were first generated in the 100 individuals from Illumina’s Omni2.5 M array intensity data using both cnvPartition (Illumina’s proprietary Beadstudio plug-in) and QuantiSNP [27], and, separately, from NGS using cn.MOPS [28], in addition to CNV calls provided by Illumina from WGS data. Altogether, there were two primary datasets called by 2 independent algorithms each, and all 4 subsequently combined for final CNV calls as described below.

Preliminary qualitative inspection of the raw distribution of all CNVs in the 100 individuals revealed 3 outlier samples with a large excess of CNV calls (Additional file 1: Figure S1). These individuals significantly skewed the average number of CNVs in the population (Additional file 1: Table S1) and were therefore removed from further consideration, yielding a cohort of 97 individuals in whom all subsequent analysis was conducted.

The 4 platforms initially identified a total of 536,889 CNVs from all 4 algorithms in the 97 individuals, including 119,236 putative deletions [copy number class (CN) 0 or 1] and 417,653 putative duplications (CN 3 or 4+; Table 1). The excess of duplications over deletions is largely a result of CNVs provided by Illumina’s proprietary WGS calls, which reported 314,656 duplications and 49,177 deletions, with no homozygous deletions (CN 0) called in the 97 samples.

**Table 1:** Copy Number Variations in the Qatari Population^a^

Parameter	Total by parameter	Homozygous deletions (CN 0)	Heterozygous deletions (CN 1)	Total deletions	Duplications (CN 3)	Amplifications (CN 4+)	Total duplications/amplifications	Total polymorphic CNVRs	Total size of all non-overlapping CNVRs in the subpopulation
Array Data									
*QSNP*	56,135	7,435	23,497	30,932	16,767	8,436	25,203	-	-
*CNVPart*	16,895	1,026	3,906	4,932	9,527	2,436	11,963	-	-
Sequencing Data									
*cn.MOPS*	100,026	2,097	32,098	34,195	50,080	15,751	65,831	-	-
*IL-NGS*	363,833	-	49,177	49,177	213,435	101,221	314,656	-	-
Total CNVs by CN Class	536,889	10,558	108,678	119,236	289,809	127,844	417,653	-	-
CNVs per individual									
*Average/individual*	1,824	120	628	748	801	275	1,076	-	-
*Median/individual*	1,815	121	622	743	801	271	1,072	-	-
*Average size by class/individual*	29,934,170	1,131,273	5,928,199	7,059,472	18,400,102	4,474,596	22,874,698	-	-
*Median size by class/individual*	27,911,587	1,087,616	5,787,942	6,875,558	16,889,655	4,146,374	21,036,029	-	-
CNV Regions (CNVRs) by genetic subpopulation									
*Q1*	5,241	149	2,534	2,683	1,480	270	1,750	808	85,705,083
*Q2*	4,176	116	1,909	2,025	1,242	273	1,515	636	65,814,099
*Q3*	4,641	101	2,283	2,384	1,316	304	1,620	637	65,851,402
Total across subpopulations	14,058	366	6,726	7,092	4,038	847	4,885	2,081	-
Average size of CNVRs within each class	15,462	4,187	8,604	8,376	20,101	10,669	18,457	32,561	-

^a^Four different algorithms were applied to detect CNVs in 97 individuals. For analysis of the Illumina Omni2.5 M Array Data, QuantiSNP (QSNP) [27] and Illumina’s cnvPartition (CNVPart) were used; for next-generation-sequencing (NGS) genomic data, cn.MOPS (CNMOPS) [28] was used with additional CNV calls provided by Illumina’s genome-sequencing service (IL-NGS). Shown are the numbers of CNVs detected by each algorithm in each copy number class, along with the total number of CNVs detected by copy number (CN) class and by CNV platform. CN (Copy number) class 0 = homozygous deletions; CN 1 = heterozygous deletions; CN 3 = single-allele duplication; CN 4 + = amplifications. Total deletions and duplications are a sum of CN classes 0, 1 and 3, 4+, respectively. Total CNVs and size are shown by platform and by class. As expected, array-based methods generated fewer but larger CNVs, whereas NGS based methods generated more but, on the average, smaller CNVs. The number of CNVs per individual is shown for the average and median individual amongst 97 individuals who passed the QC. CNV counts are shown by CN class. Additionally, the size of genomic content that is altered by CNVs in each CN class in the average and median individuals are provided. As described in Methods, these CNVs were merged across individuals within the same subpopulation to arrive at subpopulation level CNV Regions (CNVRs). The number of CNVRs within each subpopulation is given for each CN class, and the size of the average CNVR within each class is also shown. Within a population, there are sites that sometimes contain both deletions and duplications in different individuals; these are tallied in a column labeled ‘polymorphic’ CNVRs and represent about 15 % of all CNVRs within a given population. Finally, the total size of all non-overlapping CNV regions within each subpopulation is shown in the last column. The 3 genetic subpopulations are Q1 (Bedouin ancestry, *n* = 57), Q2 (Persian/South Asian ancestry, *n* = 20), and Q3 (African ancestry, *n* = 20)

In order to enhance specificity, we devised an approach to integrate CNV calls across all 4 platforms, requiring a CNV to be observed at least twice to be retained (Fig. 1). In this step, each of the 536,889 raw, high quality CNVs was first compared to all other CNVs detected by any of the four platforms within the same individual file, and those observed twice (detected by >1 algorithm in the same individual) were included in the ‘final’ variant file for that individual. All CNVs observed only once were then compared across all other individuals to look for a second occurrence in another individual. If found, that CNV was retained in the individual’s ‘final’ variant file, or otherwise discarded. This allowed for significant refinement of the list of CNVs in the population, eliminating all singleton CNV occurrences in the population – usually the most enriched for spurious calls. Using this approach, the average individual’s ‘final’ genome had 1824 high-confidence CNVs, comprising 120 homozygous deletions, 628 single-copy deletions, 801 single-copy duplications and 275 amplifications (Table 1).

During this filtration and integration process, all CNVs were concurrently curated to re-define breakpoints based on the source of CNVs. Briefly, whenever NGS-derived CNVs overlapped array-CNVs, we used the NGS’s higher-resolution breakpoints to define the start and/or end coordinate of the duplication or deletion. Wherever two CNVs detected from the same platform were observed to overlap, the narrower breakpoints were chosen, yielding a shorter, more conservative CNV call. After this curation, the ‘final’ CNV content in the average Qatari genome affected a total of 29.9 × 10^6^ non-redundant bases (Table 1). This is slightly lower than previously published estimates [22] and may reflect the strict filtration and breakpoint definition thresholds applied in this study.

The median Qatari genome, based on the four different algorithms and platforms, contained 1815 high-resolution CNVs, covering an estimated total of 27,991,857 bp (Table 1). These were distributed into the 4 CN classes: CN–0 (homozygous deletions), 121 CNVs, affecting 1.1 Mb; CN–1 (single-allele deletions), 622 CNVs, affecting 5.8 Mb; CN–3 (single-allele duplication), 801, affecting 16.8 Mb; and CN–4+ (amplification), 271, affecting 4.1 Mb. The excess number and larger size of duplications could be explained by a higher proportion of duplications detected by our NGS-CNV callers (Table 1), and may reflect a combination of reads mapping to segmental duplications and the fact that we included all multi-allelic CNVs with more than 4 copies in the same amplification group [11]. Of 1815 CNVs, 1381 (76.1 %) were detected by NGS alone, 122 (6.7 %) were detected by array technology only, and 312 (17.2 %) were detected by both, suggesting that the two datasets may both be beneficial in representing the total CNV content within an individual, and relying on only one may not be sufficient to cover all variation (Additional file 1: Figure S2).

### CNV distribution in Qatari subpopulations

The 97 individuals were examined in the context of the three Qatari ancestral subpopulations (57 Q1 – Bedouin ancestry, 20 Q2 – Persian ancestry, and 20 Q3 – African ancestry). In order to evaluate the accuracy of CNV calls, we initially used the CNVs detected across all 97 individuals and performed principle component analysis. This analysis separated individuals previously known to belong to Q1, Q2 and Q3 from genotyping data into their three respective subpopulations based on CNV sharing. The PCA plot showed some level of overlap between Q1 and Q2 clusters, which could be a result of admixture and our assignment of ethnicity based on only 65 % of 48 SNPs (Additional file 1: Figure S3 and details in methods), with Q3 (with the exception of 1 individual) being the most clearly distinct subpopulation. These results are similar to those obtained from a PCA plot using only SNPs, as published in [29]. The similarity of clustering using PCA on CNV and genotyping data in 97 Qataris is consistent with a previous report demonstrating that PCA analysis based on high quality CNVs yields similar clusters to one based on SNPs from the same individuals [30].

**Fig. 2:**
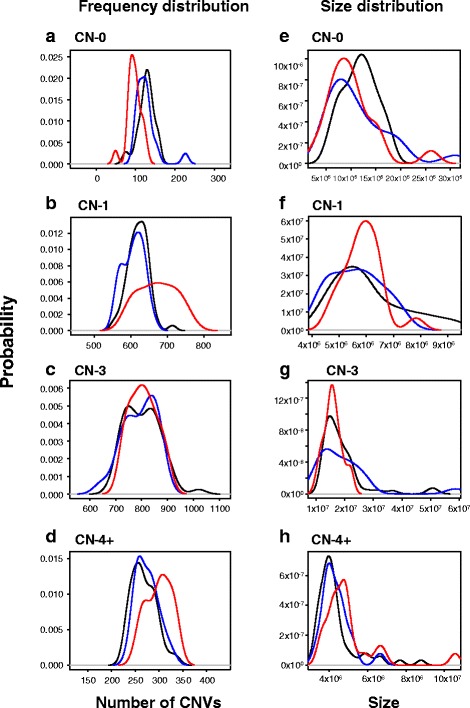
Probability distributions of CNVs by frequency and size in each copy number class in 97 Qataris. Density curves showing the probability (y-axis) of a given individual from each of the 3 subpopulations having a certain number of CNVs (**a**-**d**) or a certain cumulative size of the genome affected by CNVs (**e**-**h**) in each copy number class (**a**, **e**. CN = 0; **b**, **f**. CN = 1; **c**, **g**. CN = 3; **d**, **h**. CN = 4+). All p -values are calculated using the ANOVA-Tukey method. Black trace – Q1, Blue trace - Q2, Red trace – Q3

We then inspected the distribution of CNVs by frequency in each class per individual (Additional file 1: Figure S4), and observed that, on average, individuals from all three subpopulations had a similar range of CNVs in all four classes. However, in order to detect if the three genetic subpopulations may have differences in the distribution by number or size of CNVs in each CN class, probability curves were generated of CN number (Fig. 2a-d) and total size affected (Fig. 2e-h) within each CN class for each of the 3 subpopulations (as described in Methods). For CN class 0 (homozygous deletions), these occurred at a significantly higher frequency in Q1 and Q2 over Q3 (*p* = 1.8x10^−6^ and 1.2 × 10^−4^, respectively). However, this trend was reversed in amplifications (CN 4+), which were found at a higher rate in Q3 than either Q1 or Q2 (*p* = 1.5 × 10^−5^ and 0.006, respectively). These observations may reflect higher consanguinity rates in recent generations within Q1 and Q2, where enrichment in homozygous deletions (Fig. 2a) but depletion of amplifications *vs* Q3 (Fig. 2d) suggests that homozygous deletions are more harmful than multi-allelic, runaway duplications, and may therefore have been purged from Q3 by purifying selection over population history but only recently arisen in Q1 and Q2. This possibility is supported by two further observations. First, for single-copy deletions (CN 1), we observed a significantly higher number in Q3 (*p* = 3 × 10^−7^ and 1 × 10^−7^*vs* Q1 and Q2, respectively) despite the depletion of homozygous deletions relative to the other two subpopulations, suggesting higher diversity and less consanguinity in recent generations among Q3 Qataris *vs* Q1 or Q2. Second, for Q1, we observe a slightly longer tail in the size of the genome affected by single copy deletions (Fig. 2f) despite reduced number of CNVs in that class compared to Q3, suggesting these alleles are larger in size and possibly more recent or more deleterious, causing this tail of large CNVs to be absent in the homozygous subset of CNVs in Q1 (Fig. 2e).

### Genomic impact of CNVRs in the genetic subpopulations

In order to evaluate the impact of duplications and deletions on each subpopulation individually, we first separately merged deletions and duplications within each group to detect subpopulation-specific CNV Regions (CNVRs). There were a total of 16,660 CNVRs in the 3 subpopulations; 12,709 (76.2 %) came from NGS data only, 1976 (11.9 %) from array only, and 1975 (11.9 %) from both platforms combined (Additional file 1: Figure S2B; see Additional file 1: Additional Data). When deletions and duplications at the same locus (polymorphic CNVRs) were combined, there were a total of 14,058 CNVRs, including 7092 deletions, 4885 duplications, and 2081 polymorphic CNVRs (Table 1).

In the Q1 subpopulation, there were a total of 5241 CNVRs of all CN classes, affecting 85.7 Mb of genomic content; in Q2, 4176 CNVRs affecting 65.8 Mb, and in Q3 4641 CNVRs affecting 65.8 Mb (Table 1). The excess number and cumulative size of CNVRs in Q1 is likely due to the ~3-fold higher number of individuals studied. As expected, the majority of CNVRs were sub-population specific, with 3624, 3242 and 3633 CNVRs at low-frequency (affecting 1 to 20 % of individuals) in Q1, Q2 and Q3 respectively, *vs* only 2657, 1715 and 1789 that were common (affecting >20 %).

### Functional effect of CNV-affected genes in Q1, Q2 and Q3

In order to evaluate the functional effect of deletions and duplications separately on the entire population, the polymorphic CNVRs were separated into their respective CN classes (Table 2). In total, 16,660 CNVRs were observed in all four CN classes in the three subpopulations, including 6281 in Q1, 4957 in Q2 and 5422 in Q3. In all three subpopulations, ~39-40 % of all CNVRs were genic (2491 in Q1, 1995 in Q2 and 2085 in Q3), 4-5 % affected microRNA loci (229 in Q1, 183 in Q2 and 180 in Q3), 13-15 % affected promoter sites (831 in Q1, 647 in Q2 and 660 in Q3) and ~38-40 % affected transcription factor binding sites (2573 in Q1, 1879 in Q2 and 2065 in Q3). We focused on genic CNVs in subsequent analysis to determine the extent of CNV impact on genes and pathways and population burden for genetic disease.

**Table 2:** Functional Annotation of CNV Regions in the Qatari Genetic Subpopulations^a^

		Total number	Nongenic	Genic	miRNA	Genic affecting Mendelian disease genes	# Mendelian genes affected	Overlap DGV	Novel	Novel genic	Promoter site	Transcription factor binding site
Q1 –	0 – homozygous deletions	341	228	113	2	8	8	322	19	7	20	127
	1 – heterozygous deletions	3,316	2,151	1,165	19	76	83	3,091	225	97	248	1,321
	3 – duplication	2,161	1,121	1,040	196	182	223	2,045	116	48	509	980
	4 – amplification	463	290	173	12	18	18	445	18	5	54	145
Sub-total Q1		6,281	3,790	2,491	229	284	332	5,903	378	157	831	2,573
Q2 –	0 – homozygous deletions	293	183	110	1	11	11	287	6	1	14	109
	1 – heterozygous deletions	2,470	1,598	872	15	65	70	2,350	120	53	172	879
	3 – duplication	1,760	913	847	156	124	157	1,694	66	28	409	748
	4 – amplification	434	268	166	11	17	17	422	12	5	52	143
Sub-total Q2		4,957	2,962	1,995	183	217	255	4,753	204	87	647	1,879
Q3 –	0 – homozygous deletions	267	174	93	0	18	8	262	5	2	16	101
	1 – heterozygous deletions	2,858	1,902	956	13	61	65	2,726	132	45	176	1,046
	3 – duplication	1,835	977	858	148	122	141	1,754	81	29	407	772
	4 – amplification	462	284	178	19	23	24	443	19	9	61	146
Sub-total Q3		5,422	3,337	2,085	180	224	238	5,185	237	85	660	2,065
Total		16,660	10,089	6,571	592	725	825	15,841	819	329	2,138	6,517

^a^CNVRs were annotated as described in Methods. Distribution of CNVs is summarized by CN class within each subpopulation and by functional class including: Total number = all CNVRs detected; nongenic = CNVRs that do not overlap coding regions; genic = CNVRs that overlap genes; miRNA = CNVRs that overlap microRNAs; Mendelian disease genes = CNVRs that include at least 1 known Mendelian disease gene; DGV = CNVRs that overlap a known CNV from the database of genomic variants; novel = CNVRs that do not overlap known CNVs and are unique to Qataris; novel genic = the subset of novel CNVRs that overlap at least 1 gene; promoter site = CNVRs that overlap promoter elements; transcription factor site = CNVRs that overlap at least 1 transcription factor site. Total for each subpopulation is a sum of deletions and duplications in each subpopulation

### Genic pathway enrichment

The genes affected by CNVRs in all Qataris were evaluated by standard pathway analysis against the KEGG pathway database using the DAVID bioinformatics suite [31–33]. Among the top 15 pathways enriched for by genes affected by all CNVs in Qataris, we observed several of potential concern for public health (Table 3). These included genes involved in starch and sugar metabolism, in the insulin signaling pathway, and in type I and type II diabetes mellitus (Additional file 1: Figure S5A-E). Among these genes was the amylase enzyme AMY1, for which decreased copy number was previously shown to be associated with obesity [34]. Of interest, 47 of 97 individuals in the cohort had type 2 diabetes (27 Q1, 10 Q2 and 10 Q3), but there was no statistical enrichment for any of these CNVs in obese or diabetic individuals *vs* controls. This may be due to the low power in small sample size, combined with the possibility that individuals labeled as controls have yet to develop diabetes due to their young age at time of assessment (cohort average age 42 years, with >50 % <40 year). We also observed nominal enrichment in other medically relevant pathways, including drug metabolism and non-small cell lung cancer (Table 3). Together, these observations suggest that CNVs in this population may affect public health by contributing to the burden of chronic disease in the population and should be assessed systematically in a larger cohort to establish power and assess significance.

**Table 3:** Top 15 KEGG Pathways Enriched in Genes Affected by CNVs in Qataris^a^

Kegg pathway	Number of genes	Fold-enrichment	p value
Notch signaling pathway	16	2.7	4.4 × 10^−4^
Starch and sucrose metabolism	14	2.6	1.5 × 10^−3^
Focal adhesion	39	1.5	6.4 × 10^−3^
mTOR signaling pathway	14	2.1	1.1 × 10^−2^
Purine metabolism	30	1.5	1.6 × 10^−2^
Antigen processing and presentation	19	1.8	1.4 × 10^−2^
Axon guidance	25	1.5	3.2 × 10^−2^
Type II diabetes mellitus	12	2	3.0 × 10^−2^
Drug metabolism	14	1.8	4.4 × 10^−2^
Extracellular matrix - receptor interaction	17	1.6	6.0 × 10^−2^
Type I diabetes mellitus	10	1.9	7.6 × 10^−2^
Non-small cell lung cancer	12	1.8	7.2 × 10^−2^
Insulin signaling pathway	24	1.4	8.2 × 10^−2^
Metabolism of xenobiotics by cytochrome P450	13	1.7	7.0 × 10^−2^
Maturity onset diabetes of the young	7	2.2	8.7 × 10^−2^

^a^All genes affected by CNVs in Qataris were analyzed by DAVID bioinformatics resources for KEGG pathways. Number of genes in each enriched KEGG Pathway, along with the fold-enrichment within each pathway and a p value for the significance of enrichment

### CNVs affecting Mendelian disease genes

In order to determine whether CNVs may also play a role in rare disease in Qataris, we compared all genes affected by CNVRs to the database of Online Mendelian Inheritance in Man (OMIM). In all three subpopulations, approximately 10 % of all genic CNVRs affected at least 1 OMIM gene (Table 2). The OMIM database contains a combination of disease causing genes, as well as disease-associated genes and genes affecting polymorphic traits. Because we were most interested in genes that have sufficient evidence of disease-causality from the literature, we re-annotated all CNV-encompassed OMIM genes based on their published role in causing disease, and then manually curated all putative OMIM-gene-containing CNVRs to determine the exact number of exons that were likely to be disrupted by each CNV (contained within the CNVs’ breakpoints).

The focus was on the subset of CNVRs most likely to have a functional impact on a gene. These include deletions affecting any number of exons and duplications that either encompass at least one entire gene (increased dosage) or are internal to the gene (possibly disrupting protein translation frame). We therefore eliminated from consideration all intronic events as well as duplications that were partially genic (one breakpoint extending past the first or last exon with the other breakpoint inside the gene). We then split the list of OMIM-affected genes into two groups: (1) genes in which CNVRs had been previously reported; and (2) genes affected by novel, Qatari-specific CNVRs. In the former group, we found a total of 46 disrupted disease-causing genes (13 in deletions and 33 in duplications) affected by 40 unique CNVR loci (13 deletions and 27 duplications) (Table 4). These CNVRs had variable distribution among the 3 subpopulations, with most being specific to one or two populations while only 9 were observed in all 3 subpopulations. Thus, the majority of Mendelian-disease-gene containing CNVRs is population-specific, and may predispose to disease due to high levels of intra-population mating. Further, though these CNVRs are marked as previously reported due to overlap with CNVRs in the database of genomic variants (DGV) [35], it is possible due to the variable breakpoints of CNVs deposited in the DGV that these Qatari CNVs affect different exons or occur at a higher frequency in this population than the rest of the world.

**Table 4:** Qatari Genetic Subpopulation-specific Distribution of Known CNV Regions Deletions Affecting Known Mendelian Disease Genes^a^

Disease (MIM number)	Gene	Exons affected	CHR	Start	End	Size	Q1^2^	Q2^2^	Q3^2^
Deletion									
Age related macular degeneration (603075)	HMCN1	31/107	1q31.1	185979151	185985000	5849	-	<1 %	-
Chediak-Higashi syndrome (214500)	LYST	47/53	1q42.3	235854998	235858929	3931	<1 %	<1 %	-
Dystonia 16 (612067)	PRKRA	6-7/7	2q31.2	179296981	179300871	3890	>10 %	1-10 %	1-10 %
Glutaric acidemia IIC (231680)	ETFDH	1/13	4q32.1	159591175	159594157	2982	<1 %	1-10 %	-
Distal myopathy (606070)	MATR3	16-17/18	5q31.2	138661971	138665031	3060	1-10 %	1-10 %	<1 %
Prostate cancer (176807)	MSR1	5-10/10	8p22	15945301	16023600	78299	-	<1 %	-
Alpha-methylacetoacetic aciduria (203750)	ACAT1	2-3/12	11q22.3	108002099	108004927	2828	<1 %	-	<1 %
Keutel syndrome (245150)	MGP	1-5/5	12p12.3	15035821	15051689	15868	1-10 %	-	-
von Willebrand disease (193400)	VWF	4-5/52	12p13.31	6218203	6225614	7411	1-10 %	1-10 %	-
Adams-Oliver syndrome (614219)	DOCK6	15-28/48	19p13.2	11332570	11350981	18411	1-10 %	1-10 %	<1 %
Nephrotic syndrom (256300)	NPHS1	21-22/29	19q13.12	36328501	36331200	2699	-	<1 %	-
Bleeding disorder, platelet-type (614201)	GP6	7-8/8	19q13.42	55523566	55526400	2834	-	-	<1 %
Essential hypertension (14550)	PTGIS	9-10/10	20q13.13	48124290	48128451	4161	<1 %	-	-
Duplication									
Corneal dystrophy (136800)	COL8A2	2/2	1p34.3	36559621	36565584	5963	-	-	<1 %
Cerebellar ataxia (614756)	CAMTA1	11/23	1p36.23	7735380	7742501	7121	1-10 %	>10 %	-
Peroxisome biogenesis disorder (614870)	PEX10	1-6/6	1p36.33,p36.32	2283844	2539006	255162	>10 %	-	-
Holoprosencephaly-9 (610829)	GLI2	10-13/13	2q14.2	121739875	121747372	7497	1-10 %	-	<1 %
N-acetylaspartate deficiency (614063)	NAT8L	1-3/3	4p16.3	2035597	2071655	36058	>10 %	>10 %	1-10 %
Primary ciliary dyskinesia 3 (608644)	DNAH5	48-50/79	5p15.2	13791701	13795151	3450	1-10 %	-	-
Bone marrow failure (614742)	TERT	4-15/15	5p15.33	1230427	1255520	25093	1-10 %	-	-
Recessive spastic paraplegia (613647)	AP5Z1	1-17/17	7p22.1	4805669	4877956	72287	>10 %	>10 %	>10 %
Progressive myoclonic epilepsy (611726)	KCTD7	1-5/5	7q11.21	66071436	66132291	60855	-	-	<1 %
5-oxoprolinase deficiency (260005)	OPLAH	1-28/28	8q24.3	144773296	145216604	443308	>10 %	>10 %	1-10 %
Amelogenesis imperfecta, type 3 (130900)	FAM83H	1-5/5	8q24.3	144773296	145216604	443308	>10 %	>10 %	1-10 %
Muscular dystrophy with epidermolysis bullosa (226670)	PLEC	1-32/32	8q24.3	144773296	145216604	443308	>10 %	>10 %	1-10 %
Acrodermatitis enteropathica (201100)	SLC39A4	1-12/12	8q24.3	145278809	145771012	492203	>10 %	-	-
Rothmund-Thomson syndrome (268400)	RECQL4	1-22/22	8q24.3	145278809	145771012	492203	>10 %	-	-
Myasthenic syndrome (608931)	MUSK	2-3/13	9q31.3	113439201	113451401	12200	<1 %	<1 %	-
Autosomal dominant mental retardation (614254)	GRIN1	1-21/21	9q34.3	139887971	140232124	344153	>10 %	>10 %	>10 %
Hypophosphatemic rickets with hypercalciuria (241530)	SLC34A3	1-13/13	9q34.3	139887971	140232124	344153	>10 %	>10 %	>10 %
Recessive deafness (613307)	TPRN	1-4/4	9q34.3	139887971	140232124	344153	>10 %	>10 %	>10 %
Recessive mental retardation (614202)	MAN1B1	1-14/14	9q34.3	139887971	140232124	344153	>10 %	>10 %	>10 %
Osteogenesis imperfecta, type V (610967)	IFITM5	1-2/2	11p15.5	280817	312896	32079	-	-	1-10 %
Famililial hyperproinsulinemia (MODY) (613370)	INS	1-2/2	11p15.5	2179313	2194175	14862	1-10 %	1-10 %	1-10 %
Segawa syndrome (605407)	TH	1-14/14	11p15.5	2179313	2194175	14862	1-10 %	1-10 %	1-10 %
Primary congenital glaucoma (613086)	LTBP3	1-10/10	11q13.1	65305964	65407963	101999	>10 %	-	-
Pyruvate carboxylase deficiency (266150)	PC	13-18/22	11q13.2	66617727	66629986	12259	1-10 %	-	-
Mitochondrial myopathy and sideroblastic anemia (600462)	PUS1	1-4/6	12q24.33	132369172	132424944	55772	>10 %	-	-
GABA-transaminase deficiency (613163)	ABAT	1-16/16	16p13.2	8723887	8875529	151642	-	-	<1 %
Progressive myopathy with developmental delay (613076)	GFER	1-3/3	16p13.3	2003399	2285357	281958	>10 %	1-10 %	-
Polycystic kidney disease, adult type I (173900)	PKD1	1-46/46	16p13.3	2003399	2285357	281958	>10 %	1-10 %	-
Tuberous sclerosis 2 (606690)	TSC2	1-23/23	16p13.3	2003399	2285357	281958	>10 %	1-10 %	-
Tyrosinemia, type II (276600)	TAT	1-12/12	16q22.2	71541001	71622751	81750	-	-	<1 %
Cataract (610202)	MAF	1-2/2	16q23.2	79620742	79638078	17336	1-10 %	1-10 %	1-10 %
Huntington disease-like 2 (606438)	JPH3	2/5	16q24.2	87720933	87724383	3450	1-10 %	<1 %	<1 %
Knobloch syndrome (267750)	COL18A1	1-41/41	21q22.3	46853110	46974756	121646	-	1-10 %	1-10 %
Bethlem myopathy (158810)	COL6A1	3-35/35	21q22.3	47390167	47435702	45535	>10	1-10	1-10 %
Recessive familial candidiasis (613953)	IL17RA	1/1	22q11.1	17595746	17616510	20764	1-10 %	-	1-10 %

^a^Genes affected by CNVRs in each subpopulation were looked up in the database for Online Mendelian Inheritance in Man (OMIM) for confirmed role in disease. Disease name, MIM number (OMIM identifier) and gene appear in the first two columns, followed by CNVR-centric information and sub-population-centric data. Start-End: coordinates of CNV containing OMIM gene; Deleted/Duplicated exons: exons from each gene within the boundaries of the deletion or duplication

^2^Q1, Q2 and Q3: Percentage of individuals in each subpopulation carrying this CNVR. “-” indicates CNVR not present in this subpopulation

We also examined OMIM-gene-containing CNVRs that were novel to Qataris. To determine novelty here, Qatari CNVRs were compared to CNVRs reported in the 1000 Genomes Phase I [36] study that were detected through next-generation sequencing with high-resolution breakpoints. Only 14 Qatari CNVRs passed this filter, reflecting the high diversity of populations represented in the 1000 Genomes data. These CNVRs included 9 deletions and 5 duplications (Table 5). Five of these CNVRs were Qatari sub-population-specific, while nine were shared by 2 or more sub-populations. Of the shared CNVRs, there were four deletions – one of exon 47 in the Chediak-Higashi syndrome gene LYST (lysosomal trafficking regulator gene) observed in one Q1 and one Q2 individuals, one in the glutaric acidemia gene ETFDH (electron transfer flavoprotein dehydrogenase) in one Q1 and one Q2 individuals, one in exons 2 to 3 of the alpha-methyl acetoacetic aciduria gene ACAT1 (acetyl-coA acetyltransferase 1) in one Q1 and one Q3 individuals, and one in exons 1-7 of the Gitelman Syndrome gene solute carrier 12, family member 3 (SLC12A3) observed in one Q1 and two Q2 individuals. All of these disorders are autosomal recessive and these deletions putatively truncate the genes and therefore predispose these subpopulations to these diseases if present in homozygous state. Additionally, there was one disease-gene affecting CNVR that was present in 7 individuals from all three subpopulations (5 Q1, 1 Q2 and 1 Q3), a 3 kb internal duplication of exons 13-14 of PMS2 (post-meiotic segregation increased in *S. cerevisiae 2*), a gene in which mutations in both alleles are observed in patients with hereditary nonpolyposis cancer and mismatch repair cancer syndrome. Additionally, 3 other individuals (2 Q1, 1 Q2) had a smaller (2.7 kb) deletion affecting the same exons. In total, 10 individuals (10.3 % of the cohort) had a CNV not present in public databases that putatively disrupts PMS2. Of note, colorectal cancer is the second most common cancer in Qatari males and third most common in females [37]; whether this gene contributes to the burden of colorectal cancer in this population is currently not known.

**Table 5:** Novel Qatari-specific CNVRs Affecting OMIM Disease Genes^a^

OMIM disorder	MIM number	OMIM gene	OMIM gene name	Exons affected	Other affected genes	Type	ChrCytoband: start-end	Size (bp)	Q1 (*n* = 57)	Q2 (*n* = 20)	Q3 (*n* = 20)
Age-related macular degeneration	603075	HMCN1	Hemonectin	31/107	-	Deletion	1q31.1:185979151-185985000	5849	0	1	0
Chediak-Higashi syndrome	214500	LYST	Lysosomal trafficking regulator	47/53	-	Deletion	1q42.3:235854998-235858929	3931	1	1	0
Glutaric acidemia IIC	231680	ETFDH	Electron transfer flavoprotein dehydrogenase	1/13	C4orf46	Deletion	4q32.1:159591175-159594157	2982	1	1	0
Hereditary nonpolyposis colorectal cancer, type 4	614337	PMS2	Post-meiotic segregation increased, S. cerevisiae 2	13-14/15	-	Deletion	7p22.1:6016951-6019650	2699	2	1	0
Microcephaly 1, autosomal recessive	251200	MCPH1	Microcephaly 1	14/14	-	Deletion	8p23.1:6493670-6501582	7912	1	0	0
Deafness, autosomal dominant	608641	GRHL2	Grainy-head like 2	8/16	-	Deletion	8q22.3:102604016-102619491	15475	0	1	0
Alpha-methylacetoacetic aciduria	203750	ACAT1	Acetyl-CoA acetyltransferase 1	2-3/12	-	Deletion	11q22.3:108002099-108004927	2828	1	0	1
Gitelman syndrome	263800	SLC12A3	Solute carrier family 12, member 3	1-7/26	NUP9, miR-138-2	Deletion	16q13:56857680-56905458	47778	1	2	0
Essential hypertension	145500	PTGIS	Prostaglandin I2 synthase	9-10/10	-	Deletion	20q13.13:48124290-48128451	4161	1	0	0
Saethre-Chotzen syndrome; craniosynostosis, type 1; Robinow-Sorauf syndrome	101400; 123100; 180750	TWIST1	Twist basic helix-loop-helix transcription factor 1	1-2/2	miR-137, miR-25/32/92/92ab/363/367, miR-33/33ab, miR-543	Full duplication	7p21.1:19149966-19157073	7107	1	0	0
Tyrosinemia, type II	276600	TAT	Tyrosine aminotransferase	1-12/12	CHST4, miR-485, miR-202, miR-125/351	Full duplication	16q22.2:71541001-71622751	81750	0	0	1
Holoprosencephaly-9	610829	GLI2	Gli-kruppel family member 2	10-13/13	-	Internal duplication	2q14.2:121739875-121747372	7497	2	0	1
Hereditary nonpolyposis colorectal cancer, type 4	614337	PMS2	Post-meiotic segregation increased, S. cerevisiae 2	13-14/15	-	Internal duplication	7p22.1:6016501-6019650	3149	5	1	1
Congenital myasthenic syndrome	608931	MUSK	Muscle, skeletal receptor tyrosine kinase	2-3/14	-	Internal duplication	9q31.3:113439201-113451401	12200	1	1	0

^a^Qatari CNVRs were compared to CNVRs from the 1000 Genomes Phase I study (*n* = 1092) [25] that were generated using next-generation sequencing technologies. Only 14 CNVRs were novel, including 9 deletions and 5 duplications. OMIM disorder – name of disorder as it appears in the OMIM database. MIM number – OMIM identifier. Del/Dup – Whether CNVR is a deletion or duplication (full or internal). Other affected genes – Other genes (not in OMIM) within the same CNV. ChrCytoband:start-end – Genomic location of the CNVR in Qataris. Size – Size of CNVR. Q1, Q2, Q3 – Qatari subpopulation (n denotes number of individuals in each subpopulation)

### Qatari CNVs affecting known disease cytobands

There has been substantial evidence implicating CNV mutations in a range of diseases, including obesity, congenital heart disease and a variety of neuropsychiatric disorders [13–24, 31]. In particular, there is a growing body of literature suggesting rare but recurrent CNVs at several loci are responsible for a proportion of these diseases in sporadic cohorts [14, 18, 20, 21, 23, 38]. We sought to determine the burden of CNVs by chromosomal cytoband to detect any enrichment in Qatari Arabs over global cohorts. Because the database of genomic variants (DGV) contains >200,000 CNVRs from >200 studies [35] detected using a wide variety of low- and high-resolution platforms, we limited this comparison to CNVRs detected by an equally high-resolution platform (next-generation sequencing) in the diverse 1000 Genomes Project phase I study (1000Gp1) [36]. All CNVRs reported in the 1000Gp1 dataset and from our study in each of the 3 Qatari subpopulations were annotated by cytoband. Of 862 cytobands in the 24 human chromosomes, 769 contained CNVRs in the 1000 genomes samples; of these, 741 had CNVRs in Q1, 708 in Q2, and 735 in Q3. There were several cytobands observed in which unique CNVRs were observed at a much higher frequency (1.5 to 10 times more non-overlapping CNVRs per cytoband) in any one of the Qatari subpopulations than in the phase I data. Among the top 10 cytobands (Table 6) with the highest enrichment were several disease-associated hotspot loci, including: 1q21.1 (Q1 *p* = 1.6 × 10^−16^, Q2 *p* = 9.67 × 10^−22^ and Q3 *p* = 2.4 × 10^−20^), in which recurrent CNVs have been observed in patients with schizophrenia and congenital heart disease; 5q13.2 (*p* = 3.4 × 10^−11^, 8.2 × 10^−14^ and 1.8 × 10^−11^ in Q1, Q2 and Q3 respectively), a locus associated with neurological disorders and alcohol dependence, 16p11.2 (*p* = 7.1 × 10^−9^, 8.1 × 10^−12^; and 1.2 × 10^−11^ in Q1, Q2 and Q3 respectively), a locus highly associated with autism, schizophrenia and childhood obesity; and 1p36.33 (*p* = 2.87 × 10^−7^ in Q3 only), a locus associated with disorders of sexual development and obesity [39–43]. CNVRs in these loci may contribute to the collective burden of these disorders in the Qatari population.

**Table 6:** Top 10 Cytobands in Which Qatari Genetic Subpopulations’ CNVRs were Observed at a Significantly Higher Frequency than in 1000 Genomes Phase I CNV Data^a^

Qatari genetic subpopulation	Cytoband	1000Genomes phase I count	Q1, Q2 or Q3 count	*p* value	Diseases associated with cytoband
Q1	1q21.1	10	55	1.60 × 10^−16^	Schizophrenia; congenital heart disease
	9q21.11	5	42	1.06 × 10^−14^	-
	5q13.2	6	35	3.43 × 10^−11^	Neurologic disorders; alcohol dependence
	9p11.2	17	49	8.97 × 10^−11^	-
	16p11.2	23	50	7.10 × 10^−9^	Autism; schizophrenia; obesity
	10q11.22	7	29	2.45 × 10^−8^	-
	9p12	14	38	2.66 × 10^−8^	-
	8p11.1	2	20	7.14 × 10^−8^	-
	9p13.1	8	29	7.55 × 10^−8^	-
	7q11.1	3	20	3.66 × 10^−7^	-
Q2	1q21.1	10	59	9.67 × 10^−22^	Schizophrenia; congenital heart disease
	5q13.2	6	36	8.21 × 10^−14^	Neurologic disorders; alcohol dependence
	9q21.11	5	33	4.22 × 10^−13^	-
	16p11.2	23	51	8.07 × 10^−12^	Autism; schizophrenia; obesity
	9p11.2	17	44	1.17 × 10^−11^	-
	7q11.1	3	25	8.91 × 10^−11^	-
	8p11.1	2	20	3.77 × 10^−9^	-
	7q11.21	35	53	5.43 × 10^−9^	-
	10q11.22	7	26	8.97 × 10^−9^	-
	9p12	14	30	1.76 × 10^−7^	-
Q3	1q21.1	10	60	2.04 × 10^−20^	Schizophrenia; congenital heart disease
	9q21.11	5	37	7.23 × 10^−14^	-
	9p11.2	17	48	6.27 × 10^−12^	-
	16p11.2	23	54	1.26 × 10^−11^	Autism; schizophrenia; obesity
	5q13.2	6	33	1.83 × 10^−11^	Neurologic disorders; alcohol dependence
	10q11.22	7	29	3.10 × 10^−9^	-
	7q11.1	3	21	3.00 × 10^−8^	-
	22q11.1	7	25	1.20 × 10^−7^	-
	8p11.1	2	18	1.24 × 10^−7^	-
	1p36.33	12	30	2.87 × 10^−7^	Disorders of sexual development; obesity

^a^All CNVRs detected in Q1, Q2 and Q3 Qataris as well as all CNVRs in the Database of Genomic Variantsfrom the 1000 Genomes Project Phase I [25] data were annotated for which chromosome and cytoband they affected. Fisher’s exact test was used to evaluate enrichment or depletion of CNVRs from a specific cytoband, corrected p value for significance (<6.7 × 10^−6^)

### Determining tagging probes for Qatari CNVRs

The Illumina OMNI2.5 M array was developed as a global population-based genotyping tool to integrate variation down to minor allele frequency (MAF) >2.5 % from the diverse populations represented in the 1000 Genomes Project [36]. We first sought to estimate the utility of the Illumina OMNI 2.5 M array for studying the Qatari population. To assess the amount of “informative” SNPs on the OMNI2.5 M array in this population, we used genotypes from a total of 108 Qataris genotyped on this platform. The OMNI 2.5 M SNPs were first pruned for those that had a call in at least 90 % of the 108 individuals (i.e., at most 11 individuals with “no calls”), which resulted in 2,368,880 (>99.5 % of all SNPs). Surprisingly, of these, 412,839 SNPs (17.4 %) were monomorphic (MAF = 0 %) in 108 Qataris. Additionally, 676,116 (28.5 %) had a MAF of less than 1 % in Qataris, and 1,028,842 (43.4 %) have a minor allele frequency of less than 5 %. Therefore, in total less than 60 % of the SNPs on the OMNI 2.5 M array adequately sample common variants in Qataris (Additional file 1: Table S2).

**Fig. 3:**
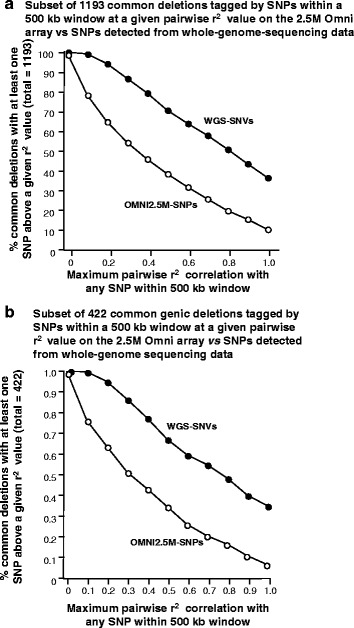
All SNPs within 500 kb of start and end breakpoints of 1,193 deletions were used to detect for each deletion a SNP with the maximum pairwise LD correlation. This was done both for **a.** all 1193 CNVs and **b.** for only 422 Genic CNVs. In both cases, the WGS SNVs significantly outperformed the OMNI2.5 M SNPs, especially at higher r^2^ values. WGS-SNVs: Whole genome sequencing detected variants (●). OMNI2.5 M-SNPs: SNPs present on the OMNI2.5 M array (Ο)

Nevertheless, we attempted to assess whether a subset of these SNPs tag common CNVs in this population, which could be useful for imputation of Arab-population CNVs from genetic data using this or similar arrays in future studies. In order to increase specificity, we focused on 1193 common deletions (deleted allele observed at least 4 times) across the 97 individuals in this study, and investigated the pairwise correlation between the deletion CNV and all SNPs within 500 kb either side of the deletion’s breakpoints. As expected, while 1168 CNVs (98 %) had at least 1 SNP within 500 kb of either breakpoint, the majority of SNPs from the OMNI 2.5 M array neighboring CNVs did not adequately tag the deletion allele, with the majority of SNPs (~62 %) having a maximum r^2^ < 0.5 (Fig. 3a). In fact, only 318 of 1193 deletions were tagged by at least one SNP at a Pearson correlation of r^2^ > 0.7, of which only 195 (~16 %) were tagged at r^2^ > 0.9 (Additional file 1: Table S3). Further, of the 422 genic subset of CNVs within the 1193 deletions, only 35 % were tagged by an array SNPs with a correlation r^2^ > 0.5, and less than 7 % appeared in complete LD (r^2^ > 1.0). Therefore, the majority of deletions common to the Qatari population were poorly tagged by the high density OMNI 2.5 M array, which could pose significant challenges to using this or other commercial arrays to genotype CNVs in Arab populations.

In order to rectify this issue, we sought to determine a set of SNPs that could better tag these CNVs by relying on genotypes obtained from the whole genome sequencing of these 97 individuals (described in methods). All ~21 million high-quality variants detected in 97 individuals were first pruned for those within 500 kb upstream and downstream of each CNV breakpoint, and then LD measured between each CNV and all neighboring SNPs within this window. There was a highly significant improvement of up to 250 % for all CNVs and almost 1.5 times that (367 %) for genic CNVs at r^2^ = 1 (Fig. 3b and Additional file 1: Table S3). With whole genome sequencing SNPs, we observed >70 % of all deletions being tagged by at least one SNP at an r^2^ > 0.5, and over 50 % at r^2^ > 0.8, suggesting these could be imputed in future experiments from sequence data. In order to facilitate the design of new genotyping arrays that tag CNVs in this population, we include a list of deletion-tagging genotypes at SNPs tagging 806 CNVs at r^2^ > 0.5 (Additional file 1: Table S4). We also include this information at greater detail in the accompanying Additional file 2 containing the complete CNV dataset with all functional annotation in 97 Qataris.

## Discussion

This study uses two primary datasets called by four separate algorithms to generate the first catalog of high-resolution copy number variants within Qataris, a population that shares significant genetic ancestry with the neighboring populations of the Arabian Gulf who remain under represented in public databases. Our analysis reveals several notable features of copy number changes in this region of the world. First, CNV distribution patterns among members of the different Qatari subpopulations is concordant with their cultural and demographic histories, where higher consanguinity in Q1 and Q2 populations has led to a significantly higher number of homozygous deletions *vs* the more diverse Q3 individuals. Notably, these deletions are larger in size, and may be useful for studies of the effect of gene-loss on individual fitness, similar to studies of loss of function mutations in large but seemingly healthy cohorts. Further, a large proportion of CNVRs across all 3 Qatari subpopulations affect coding or functional elements (with slight depletion in Q3 *vs* either Q1 or Q2), including known rare, severe disease genes and loci. Of particular interest is the increased burden of CNVs in certain KEGG pathways relevant to population health in Qatar and in the region, such as diabetes, insulin signaling and metabolism.

While all 97 individuals analyzed here are phenotypically “control” adults, the possibility that the CNVs they carry might be associated with disease cannot be ruled out. CNVs have been shown to play a role both in population diversity and in pathophysiology, where increased or decreased gene dosage may be responsible for human phenotypic variability as well as complex behavioral traits and disease (reviewed in [17, 44–46]). Indeed, we observe a significantly higher burden of CNVs in cytobands linked to known, rare syndromic disorders. Thus, the Qatari population could be at risk for any of these CNV-related disorders under a two-hit model, where incomplete penetrance and variable expressivity may depend on a second insult, possibly a point mutation, at an interacting locus [47]. Further, we found a number of CNVs that were shared within the Qatari population (but novel to public databases) affecting severe Mendelian disease causing genes, including recurrent hits (both deletions and duplications) in PMS2, a gene that is often mutated in colon cancer, the second most prevalent cancer in Qatar [37]. These observations will be informative in the design of the next generation of clinical copy number arrays for use with this population.

A distinctive feature of this study is in combining high-resolution CNV calls from whole genome sequencing with the traditional CNV calls from genotyping array data, which demonstrated a non-trivial number of CNVs detected exclusively by only one platform. This held true despite high specificity thresholds implemented to reduce spurious calls, suggesting that relying on only one of these platforms risks eliminating a substantial amount of the underlying variation. This hybrid strategy uncovered approximately 1815 CNVs of size range 2.5 kb to 2 Mb in the median Qatari individual, affecting a total of ~28 Mb of genomic DNA. This is slightly lower than previously published high-resolution studies that estimate CNVs affect up to 40 Mb of genomic DNA [10, 25], and may be due to a combination of our higher minimum size thresholds (2.5 kb vs 500 bp used in other studies) and the strict specificity criteria we employed, which eliminated all singletons. Nevertheless, we observed that up to 40 % of CNVRs affected genic content, suggesting these could contribute to key traits yet to be fully understood in the general population.

As an additional analysis, we determined the usefulness of the OMNI2.5 M array to genotype CNVs in this population. The performance of this array was relatively poor, with <35 % of CNVs tagged at r^2^ > 0.5. We decided instead to leverage the genotypes obtained from whole genome sequencing of these same individuals and found substantial improvements to discovering tagging SNPs for these population-level deletions (included in the whole CNV dataset released as Additional Data accompanying this study). These SNPs could be included in future arrays designed to genotype Arab cohorts, and used to impute these deletions in Qatari or ancestrally similar Gulf Arabs.

This study, with its small population size, is underpowered to discover any significant effect of CNVRs on common disease in the population. However, it provides the first step in creating a database of Qatari-specific CNVRs that sets the landscape for future research to assess rare and common CNVs in a much larger Qatari cohort. We suspect that the Qatari population’s unique burden of rare and chronic disease will provide a strong platform for discovery of functional CNVs in future studies. This is especially critical, for example, in the burden of deletions disrupting known disease genes (e.g. those in OMIM), which may appear in homozygous state in the highly consanguineous Qatari population. Indeed, of particular interest for human disease studies is the significantly higher proportion of homozygous deletions in Q1s and Q2s, which could shed light on the subset of the genome that is “dispensable” for normal human development into adulthood due to loss of gene function [48–50]. In this study, we find 200 genes that are affected by homozygous deletions, yet appear to cause no overt phenotypic abnormalities in this group of 97 seemingly healthy adults.

In recent years, each of genotyping arrays, array-CGH microarrays, and next-generation sequencing have all been implemented successfully to detect structural variation in human cohorts (reviewed in [45]). While the majority of studies continue with HapMap cohorts, there has been a recent increase in the number of reports from non-HapMap populations, the majority of which are still ancestrally similar to pre-existing HapMap populations (e.g., East Asian populations) [51–53]. To date, however, there has been no systematic characterization of CNVs in Gulf Arabs, a population witnessing a growth in clinical genomics testing but whose ancestry is not adequately represented in current HapMap and 1000 Genomes populations. We present this study as the first systematic approach that makes available data on a large number of CNVs detected from both genotyping and sequencing platforms in this previously under-explored population, and believe the frequency of CNVs reported in this study could be used to assess the pathogenicity of clinically detected CNVs in Gulf Arab patients that appear novel to public databases. Thus, this high-resolution map of CNVs in Qataris is likely representative of ethnically similar Gulf-Arabs, and the accompanying database will inform future studies with larger cohorts aimed at understanding the unique population history in this region and the interpretation and analysis of clinically-observed CNVs in patients.

## Conclusions

Overall, there is little doubt that some proportion of disease burden in certain patient cohorts can be explained by CNVs [17, 44–46]. As detection methods continue to improve, so will the discovery of new genes and loci whose deletion or duplication could lead to morbid clinical phenotypes in specific populations. This kind of population specificity will have detrimental effects on the design of population-specific clinical CHG-arrays, and on the interpretation and assignment of pathogenicity of CNV findings in individuals from different ethnic populations [25, 51–54]. This issue is of special relevance to Arabs in general and Qataris specifically, for whom there is a paucity of databases of CNVs observed in healthy controls.

In this study, we generate a highly dense catalog of 16,660 CNVRs in a cohort of 97 Qataris, by employing strict QC criteria for detection of CNVs and by integrating data from both SNP arrays and NGS technologies to achieve high-resolution breakpoint annotation for most variants. We find that ~40 % of these CNVRs affect genic and functional content, and that >5 % of all CNVs discovered are novel when compared to the well-populated Database of Genomic Variants [35], and thus represent polymorphisms in this population that may be misinterpreted as disease-causing in clinical cases in the absence of population-matched controls. Interestingly, despite the small sample size, we observe genes affected by these CNVRs that function in biological pathways relevant to population health, including Mendelian disease-causing genes reported in OMIM.genes involved in diabetes and carbohydrate metabolism, genes implicated in cancer, as well as homozygous deletions affecting up to 200 unique genes in seemingly healthy individuals. These are likely underestimates resulting from our strict quality filtration criteria (e.g., eliminating all true singletons and annotating breakpoints conservatively whenever two CNVs overlapped). Nevertheless, these findings pave the way for follow-up studies in larger cohorts with sufficient power and phenotypic information to determine their true contribution to disease burden in this population. Finally, we leverage the genotypes obtained from next-generation sequencing to identify SNPs that are at high linkage disequilibrium with sub-population-specific deletions, allowing their imputation in future studies of matched cohorts. This study therefore complements pre-existing and future next-generation sequencing work in Qataris, and presents findings which improve our understanding of CNV prevalence as a class of previously underexplored variation in this population, and their contribution to disease in Qataris, and possibly in ethnically similar Gulf Arabs.

## Methods

### Study population sample preparation and sequencing

In order to catalog CNVs in the Qatari population, the genomes of 108 Qataris were deeply sequenced (mean depth 37X) on the Illumina platform. Human subjects were recruited and written informed consent was obtained at Hamad Medical Corporation (HMC) and HMC Primary Health Care Centers in Doha, Qatar under protocols approved by the Joint Institutional Review Boards of Hamad Medical Corporation and Weill Cornell Medical College in Qatar (protocol # 13-00063). The population of Qatar includes over 2 million inhabitants, comprised of approximately 300,000 nationals with roots in Qatar predating the discovery of oil and gas, and establishment of an independent nation in 1970 and the over 1.7 million immigrants who mostly arrived in the past decade [55]. As selection criteria for this study, we required that subjects be third generation Qataris where all ancestors were Qatari citizens born in Qatar, as assessed by questionnaires.

#### Cohort selection criteria

Qataris with three or more generations of ancestry can be divided into 3 genetic subpopulations that reflect the historical migration patterns in the region: Q1 (Bedouin), Q2 (Persian-South Asian) and Q3 (Sub-Saharan African) [2–4]. Proportions of Q1, Q2, and Q3 ancestry were determined for each Qatari based on TaqMan genotypes (Life Technologies, Carlsbad, CA) for a panel of 48 ancestry informative SNPs [4]. A STRUCTURE analysis of population structure with k = 3 applied to the genotypes was used to estimate the proportion of ancestry in each of 3 groups (Q1, Q2, Q3). 100 individuals >65 % Q1, Q2 or Q3 were selected for genome sequencing. An additional 8 admixed Qataris who could not be cleanly placed in one of the three groups were also selected for sequencing.

#### Next-generation sequencing

Next-generation sequencing was conducted using a sequencing library preparation method that eliminates the need for size selection after shearing and PCR amplification before ligation of sequencing adapters. Sequencing was conducted at the Illumina Genome Services sequencing facility using the HiSeq 2500. Sufficient paired-end 100 bp reads were generated in order to produce a median of 112 GB of sequence data passing filters and aligned to the hg19/GRCh37 human reference genome with a median insert size of 301 bp, where at least 85 % of bases ≥ Q30, passed filtering steps and were aligned.

#### Single nucleotide variant calling

In order to identify SNPs in LD with CNVs in Qatar, SNP genotypes were generated for each Qatari genome. The Illumina Genome Network generated variant calls for the autosomal chromosomes for each of the 108 Qatari genomes using the ELAND/CASAVA v1.9 pipeline. In order to maximize confidence in the observed variants, sequence reads were re-mapped and genotypes were re-called using an in-house population genotyping pipeline [5]. Reads were realigned to the 1000 Genomes Project version of the hg19/GRCh37 human reference genome using BWA 0.5.9 [56] (maximum insert size 3 kb), and mapped reads were prepared for variant calling using GATK best practices, including PCR duplicate removal using SAMTOOLS [57], producing an average of 37x depth in autosomal chromosomes, with a mean of 98 % of mappable sites covered per genome. In order to maximize computational efficiency and integrated call set quality, calling for the autosomes was restricted to the biallelic SNPs in the combined set of 21 million segregating autosomal SNPs observed at least once in 108 Qatari genomes by the CASAVA pipeline. SNP genotypes were called for all 108 Qatari genomes using GATK [58] as described in the “Best practices for variant detection v3” Broad Institute pipeline [http://www.broadinstitute.org/gatk/]. Genotypes for each Qatari were also generated using the Illumina OMNI2.5 M array using Illumina’s GenomeStudio application.

### Copy number variant calling and analysis

Copy number was estimated from two independent primary data sets generated from the same set of 100 samples: (1) array hybridization on Illumina’s OMNI2.5 M platform; and (2) whole genome sequencing. See Additional file 1: Supplemental Methods for details on data preparation and quality control.

#### CNVs called from genotyping and Next Generation Sequencing (NGS) data

To comprehensively assess the copy number content of the 100 genomes, CNVs were assessed using four separate algorithms, two from genotyping and two from NGS data (Fig. 1). To identify CNVs from SNP genotype intensities, two calling platforms were used: Illumina GenomeStudio’s proprietary cnvPartition software, and QuantiSNP v2.0 [27], reviewed in Pinto et al. [59]. To identify CNVs from NGS, CNV calls were provided by Illumina Genome Network, and also independently called using cn.MOPS, which is suited for multi-sample CNV detection in population-level data [28]. Preliminary QC and minimum specificity thresholds for CNV calls are described in greater detail in Additional file 1: Supplementary Methods. In total, 97 samples passed the strict quality thresholds and were used for all downstream analyses.

#### Generating probability density curves of CNV distributions in the 3 subpopulations

In order to generate a probability density curve for each of the three subpopulations, we first plotted a histogram with the number of individuals having a number of CNVs or cumulative size of CNVs within a given bin. Then, using the “density” package in R (by calling the function *density(*x*)*, where x is the CNV frequency or size bin), the empirical histogram was transformed into a curve that best fits the shape of the data, and accurately approximates the probability that a given individual has a certain number or size of CNVs. The empirical data is therefore transformed into a probability density curve, in a manner similar to the results obtained using the histogram function (hist(x,probability = TRUE) for getting a probability histogram and then fitting a curve to it.

### Merging population-level CNV regions

To determine population-level CNV Regions (CNVRs), the population of 97 Qataris were first separated into their respective subpopulations (57 Q1, 20 Q2, and 20 Q3) using a panel of 48 SNPs previously as described [2–4]. The CNVs from individuals within each subpopulation were then merged separately to generate subpopulation-level CNVRs. Details of merging criteria and breakpoint assignment are found in Additional file 1: Supplementary Methods.

### CNVR annotation and data analysis

#### Annotating CNVR features

Subpopulation-specific CNVRs were annotated using Anntools (version 1.1), in which the following genomic features were annotated against genome build 37 (hg19): cytobands, genes (and affected exons), microRNAs, transcription factor binding sites and promoter sites. All genes were also custom-annotated against the Online Mendelian Inheritance in Man (OMIM) database and manually curated by critically evaluating the primary literature to differentiate disease-causing genes from disease associated genes and polymorphisms. Disease-causing genes were further annotated manually to check exactly which exons were encompassed within each CNV’s breakpoints.

#### Determining CNVR novelty

CNVR novelty was assessed by comparison to the database of genomic variants (DGV). In order to compare CNVRs discovered in Qataris with those in the DGV, DGV CNVRs were first pruned to keep only high-resolution studies, matching or exceeding the resolution of CNVs discovered in this study (2.5 M SNP arrays or next-generation sequencing). This allowed for higher confidence in breakpoint assignment and in deciding whether a CNV in Qataris was observed previously or not.

#### CNVR cytoband enrichment

For assessment of cytoband enrichment, CNVRs in each Qatari subpopulation were compared to CNVRs detected from the 1000 Genomes Phase I study, representing a very high-resolution dataset. CNVRs from that study were first annotated by the cytoband in which they occurred, and then a basic count was done for the number of unique CNVRs in each cytoband. Enrichment in the number of CNVRs affecting a given cytoband in Qataris *vs* in the 1000Genomes dataset was calculated by Fisher’s test.

#### CNVR tagging analysis

In order to determine which SNPs on the OMNI 2.5 M array adequately tag CNVRs observed in the Qatari population, we selected CNV deletions that were observed at least 4 times in the population of 97 (MAF ~2 %). The CNV genotypes were converted to binary format along with all genotypes for use by PLINK [60]. The PLINK-pairwise-ld command was used to calculate Pearson’s correlation between the genotype of each CNV and all neighboring SNPs ± 500 kb of the CNV’s breakpoints across 97 individuals. For each CNV, the closest SNP with the highest r^2^ value within the 500 kb window was identified.

Similarly, tagging SNPs from the whole genome sequencing data were identified for these same deletions. Whole genome sequencing data from all 97 individuals were obtained from Illumina PE 100 Sequencing (as described above). These were pruned for variants within 500 kb upstream and downstream of each CNV, and then plink was used as before to determine SNPs with the highest Pearson correlation (maximum r^2^ value) within these windows.

## Availability of supporting data

The data sets supporting the results of this article are included within the article and its additional files.

## Additional files

Additional file 1:
**Additional Methods, Tables, Figures and Legends.** (PDF 1401 kb) Additional file 2:
**Complete dataset (with full annotation) of all CNV regions detected in 97 Qataris.** (XLSX 3202 kb)

## Abbreviations

Array-CGH: Array-comparative genome hybridization
CN: Copy number class
CNV: Copy number variant
CNVR: Copy number variable region
LD: Linkage disequilibrium
MAF: Minor allele frequency
NGS: Next-generation sequencing
PCA: Principle component analysis
SNP: Single nucleotide polymorphism
Q1: 2, 3, Qatari subpopulation 1, 2 and 3
WGS: Whole genome sequencing
1000G: One thousand genomes project
DAVID: Database for annotation, visualization and annotated discovery
DGV: Database of genomic variants
HapMap: International haplotype map project
KEGG: Kyoto encyclopedia of genes and genomes
OMIM: Online mendelian inheritance in man
