# Supplementary material for: Copy number variations in the genome of the Qatari population
Source: BMC Genomics. 2015 Oct 22;16:834. doi: 10.1186/s12864-015-1991-5 (PMC4618522; doi:10.1186/s12864-015-1991-5)
Supplement: Additional file 1: — Additional Methods, Tables, Figures and Legends. (PDF 1401 kb) [file 12864_2015_1991_MOESM1_ESM.pdf]

Additional Data. The total dataset of 16,660 CNVs discovered in this population, including breakpoints, all functional annotation, frequencies in each subpopulation and those that are tagged by neighboring SNPs is provided.

Supplementary Methods

Primary Data Extraction and Preparation for CNV Calling

The OMNI2.5M array hybridization intensity data was preliminarily loaded into Illumina's GenomeStudio suite (V2011.1). The software normalizes hybridization intensity data, generates call rates, and determines genotypes at all SNP probe positions throughout the sample. All 108 samples passed the QC cut-off with call rates >98.5% (mean 99.7 ± 0.1% standard deviation). Using the GenomeStudio Final report wizard, final reports were exported containing hybridization intensity, allele frequencies and allele calls for all probes on the array and loaded into QuantiSNP for CNV calls as described further.

For whole genome sequencing data, all samples passed quality cut-off with mean depth > 37x. BAM files for each sample were obtained directly from Illumina's Genome Network sequencing service and loaded into cn.MOPS [1] for CNV calls as described below.

Preliminary QC and Specificity Thresholds for CNV Calls

CNVs generated from genotyping data using cnvPartition or QuantiSNP were first filtered by a minimum window size to eliminate small and possibly spurious CNVs. For homozygous deletions, a minimum window size of 2 consecutive probes was used; for all other classes, a minimum window size of 6 consecutive probes called in the same copy number class was used. For NGS-derived CNV calls, cn.MOPS uses windows of 450 bp to call CNVs. In order to enhance specificity, we required a minimum of 5 consecutive windows, yielding a minimum size of 2.25 kb. Illumina NGS-provided CNVs were also filtered for a minimum size of 2 kb, eliminating thousands of smaller CNV calls across the 100 samples. All CNVs passing these cut-offs called by all four algorithms were combined into raw individual CNV files.

Once the individual files were generated, they were evaluated to ensure that no outlier samples were carried further into the analysis. For this step, we examined the distribution of all

raw CNVs by copy number class in each of the 100 individuals, plotting their total number and total size of genome affected. Three Qatari genomes (all Q1) had a highly significant excess of CNVs (Supplemental Figure S1), and were removed from further analysis. A total of 97 Qatari genomes (57 Q1, 20 Q2 and 20 Q3) passed this QC assessment, and were taken into the population-level CNV analysis steps.

As a further QC assessment, we employed a “recurrence” filter to improve CNV specificity confidence. First, within each individual, raw CNVs from all four different platforms were checked for overlap with each other by at least 50% to detect CNV intervals that were detected by more than one algorithm. CNVs that passed this comparison (were called by more than one algorithm) were kept, while those that failed this step were then compared across the entire population, this time at 80% overlap, to detect a second occurrence of the same CNV in another individual. If found, the CNV was kept. Otherwise, CNVs that failed both steps (were only observed a single time in the entire dataset) were considered possibly spurious and discarded. Thus, for each individual, we generated a final sample file that only contained non-overlapping CNVs that were observed at least twice in the entire population. We refer to this in the results as the ‘final’ CNV file per sample.

Supplemental Table S1. Summary of "Raw" CNVs Detected in the Median and Average Individual in 100 Qataris by CN Class and Total Size[1]

CN Class	Average per individual	Median per individual	Average size by class	Median size by class
0	117	119	977,941	949,511
1	1687	669.5	14,765,937	6,649,864
3	921	809.5	18,500,812	14,000,574
4+	276	271	3,984,745	3,893,939
Total number	3001	1869		
Total size			38,249,435	25,493,888

[1] Count of all CNVs detected by all algorithms prior to QC and filtering ("Raw" CNVs), summarized as the average and median number of CNVs in each CN (copy number) class among all 100 individuals. The large deviation of the average from the median is due to 3 outlier samples which were removed from further analysis. Details of parameters used for each algorithm appear in Methods. CN Classes 0, 1, 3, 4+ represent homozygous deletions, heterozygous deletions, duplications and amplifications, respectively. Total number of raw CNVs per individual and total size of genomic content affected by all CNVs in the average and median individual also appear on the bottom. On the right, the average and median sizes of CNVs of a given class in all individuals appear.

Supplemental Table S2. Only Half of the SNPs on Omni 2.5M Array are Common and Therefore Suitable for Genotyping and Imputation in Qataris[1]

MAF in Qatari cohort	Number of SNPs (%)
0% (monomorphic)	412,930 (17.4%)
0-1%	263,816 (11.1%)
1-5%	352,726 (14.9%)
>5%	1,340,038 (56.6%)

[1] MAF: Minor allele frequency for a given SNP. Number of SNPs: a count of how many SNPs have a given MAF or MAF range. Percentage (%) is calculated as number of SNPs in a given MAF bin divided by the total number of SNPs on the Omni 2.5 for which there was a call in at least 90% of 108 individuals (2,368,880).

Supplemental Table S3. Number of CNVs Tagged by at Least One Nearby SNP at or Above Each Given Pairwise r^2 Value in the WGS-SNVs or OMNI-2.5M SNPs Datasets[1]

Category	Pairwise maximum r^2	WGS-SNVs	OMNI2.5M SNPs	% improvement of WGS over array
All CNVs	0	1193	1168	2
	0.1	1171	802	46
	0.2	1102	590	87
	0.3	1006	476	111
	0.4	914	410	123
	0.5	806	343	135
	0.6	723	303	139
	0.7	649	270	140
	0.8	566	224	153
	0.9	481	165	192
	1	400	112	257
Genic CNVs	0	420	405	4
	0.1	419	296	42
	0.2	399	196	104
	0.3	363	157	131
	0.4	318	127	150
	0.5	276	106	160
	0.6	239	90	166
	0.7	220	78	182
	0.8	184	59	212
	0.9	153	42	264
	1	131	28	368

[1] For each CNV, all SNVs or SNPs within 500kb upstream and downstream of each CNV’s breakpoints were queried for pairwise Pearson’s’ correlation with the deletion allele. For each deletion, the maximum r^2 SNP was retained. All CNVs include all 1193 deletions tested, whereas Genic CNVs include the subset of All CNVs that affects genic content. Percentage improvement of WGS over Array is calculated using ((WGS-Array)/Array*100%).

Supplemental Table S4. Genotype at 197 WGS Genomic SNPs with r^2>0.75 Tagging a Genic Deletion CNV[1]

CNV-ID	ChrCytoband	Start	End	Size	Gene(s)	TAG-Location	MAX r^2	TAG GT
cnv_675	10p11.23	31246651	31252050	5399	ZNF438	Chr10:31218620	1.00	A
cnv_694	10q11.22	46851751	46919250	67499	FAM35BP	Chr10:46712915	1.00	T
cnv_705	10q11.23	51825151	51830550	5399	FAM21A, FLJ31813	Chr10:51366738	1.00	T
cnv_707	10q11.23	51871951	51895350	23399	FAM21B, FAM21A	Chr10:51861184	1.00	G
cnv_711	10q21.3	65108251	65114550	6299	JMJD1C	Chr10:64903938	1.00	C
cnv_714	10q22.1	71230951	71238150	7199	TSPAN15	Chr10:71248553	1.00	A
cnv_717	10q22.3	78255451	78261019	5568	C10orf11	Chr10:78231092	1.00	C
cnv_759	11p11.12	49709701	49757850	48149	LOC440040	Chr11:49802029	1.00	A
cnv_734	11p15.4	3238651	3244086	5435	MRGPRG, MRGPRG-AS1	Chr11:3232953	1.00	A
cnv_740	11p15.4	5873851	5883750	9899	OR52E8	Chr11:5860035	1.00	C
cnv_741	11p15.4	7811101	7833600	22499	OR5P2	Chr11:7348902	1.00	C
cnv_780	11q12.3	63197918	63202950	5032	MIR3680-1, MIR3680-2	Chr11:63165776	1.00	C
cnv_788	11q14.3	89620201	89626500	6299	MIR5692A1	Chr11:89693373	1.00	A
cnv_792	11q14.3	89775001	89789400	14399	TRIM49C	Chr11:89819841	1.00	C
cnv_819	12p13.2	10581751	10596600	14849	KLRC2	Chr12:10498676	1.00	A
cnv_823	12p13.2	12531151	12542400	11249	LOH12CR1	Chr12:12551203	1.00	G
cnv_810	12p13.33	866701	874800	8099	WNK1	Chr12:875786	1.00	T
cnv_856	12q21.31,q21.32	86695651	86703300	7649	MGAT4C	Chr12:86680215	1.00	C
cnv_857	12q23.1	99793801	99802800	8999	ANKS1B	Chr12:99737259	1.00	A
cnv_863	12q24.32	128424938	128431350	6412	LINC00507	Chr12:128423223	1.00	G
cnv_872	13q13.1	32532301	32539050	6749	EEF1DP3	Chr13:32544447	1.00	T
cnv_873	13q13.2	34135651	34145100	9449	STARD13	Chr13:34134101	1.00	T
cnv_874	13q13.3	35558551	35564872	6321	NBEA	Chr13:35463320	1.00	C
cnv_889	13q21.33	70401966	70407000	5034	KLHL1	Chr13:70396955	1.00	T
cnv_905	14q11.2	19485451	19768050	282599	POTEG, LINC00516, LOC101101776	Chr14:19991987	1.00	C
cnv_918	14q21.3	47965051	47971350	6299	MDGA2	Chr14:47760625	1.00	G
cnv_920	14q23.3	66069001	66078000	8999	FUT8	Chr14:65879277	1.00	A
cnv_923	14q24.3	74239651	74247300	7649	ELMSAN1	Chr14:74237117	1.00	G
cnv_925	14q31.1	80105851	80115049	9198	NRXN3	Chr14:80102233	1.00	G
cnv_938	15q11.2	20934901	21318300	383399	NBEAP1, POTEB, POTEB2, NF1P2, CT60	Chr15:21372903	1.00	C
cnv_953	15q13.2	30699451	30880800	181349	ULK4P1, ULK4P2	Chr15:30656489	1.00	T
cnv_951	15q13.2	30389851	30428100	38249	ULK4P3, GOLGA8T	Chr15:30748175	1.00	C
cnv_969	15q25.2	84840751	84868200	27449	LOC100505679, LOC388152, LOC440300	Chr15:84958869	1.00	T
cnv_966	15q25.2	82957951	82965150	7199	LOC727751, LOC80154	Chr15:82660190	1.00	G
cnv_992	16p11.2	28604536	28618740	14204	SULT1A1, SULT1A2	Chr16:28625491	1.00	T
cnv_1025	16q24.3	90130951	90137250	6299	PRDM7	Chr16:90138005	1.00	A
cnv_1043	17q12	35689051	35694900	5849	ACACA	Chr17:35440342	1.00	A
cnv_1041	17q12	34725601	34759350	33749	TBC1D3H	Chr17:34588029	1.00	T
cnv_1053	17q21.31	44467201	44474850	7649	NSFP1	Chr17:44394562	1.00	T
cnv_1062	17q24.3	70815151	70821450	6299	SLC39A11	Chr17:70831364	1.00	G
cnv_1082	18q22.2	67207951	67217456	9505	DOK6	Chr18:67219917	1.00	G
cnv_1091	19p13.2	12039301	12046050	6749	ZNF700	Chr19:12053196	1.00	T
cnv_1102	19q13.12	35660701	35666100	5399	FXYD5	Chr19:35666355	1.00	G
cnv_1105	19q13.12	36840601	36847350	6749	ZFP14	Chr19:36755318	1.00	C
cnv_1109	19q13.2	40387951	40401450	13499	FCGBP	Chr19:40369435	1.00	T

Supplemental Table 4. Genotype at 197 WGS Genomic SNPs with r^2>0.75 Tagging a Genic Deletion CNV[1] (cont., page 2)

CNV-ID	ChrCytoband	Start	End	Size	Gene(s)	TAG-Location	MAX r^2	TAG GT
cnv_1111	19q13.2	43252201	43357950	105749	PSG8, PSG10P, LOC100289650	Chr19:43247881	1.00	A
cnv_1117	19q13.33	50554351	50561372	7021	FLJ26850	Chr19:50594192	1.00	T
cnv_1124	19q13.42	54800551	54807750	7199	LILRA3	Chr19:54797848	1.00	T
cnv_28	1p13.3	109496251	109501650	5399	CLCC1	Chr1:109495387	1.00	C
cnv_20	1p31.3	66024173	66030267	6094	LEPR	Chr1:66037481	1.00	T
cnv_6	1p36.21	12898801	12909390	10589	LOC649330, HNRNPCL1	Chr1:13120235	1.00	C
cnv_8	1p36.21	13105351	13136400	31049	PRAMEF5	Chr1:13159601	1.00	C
cnv_3	1p36.33	587251	623700	36449	OR4F29, OR4F3, OR4F16	Chr1:547042	1.00	T
cnv_42	1q21.1	144337051	144366300	29249	LOC100288142, LINC00623, LOC728875, PPIAL4B, PPIAL4A, PPIAL4C	Chr1:144382008	1.00	A
cnv_46	1q21.1	146356651	146365200	8549	NBPF10, LOC100288142	Chr1:146441072	1.00	G
cnv_44	1q21.1	145294860	145301156	6296	NBPF10, NBPF9, LOC100288142	Chr1:145318633	1.00	G
cnv_49	1q21.2	149313601	149459400	145799	FCGR1C	Chr1:149562286	1.00	T
cnv_48	1q21.2	149035501	149277600	242099	NBPF23	Chr1:148646826	1.00	C
cnv_56	1q25.1	174796201	174802050	5849	RABGAP1L	Chr1:174791346	1.00	T
cnv_57	1q25.1	175070701	175077000	6299	TNN	Chr1:175078475	1.00	T
cnv_64	1q32.3	213002101	213013800	11699	C1orf227	Chr1:212904544	1.00	C
cnv_73	1q44	248808151	248813550	5399	OR2T27	Chr1:248639356	1.00	A
cnv_1132	20p12.1	14271751	14279463	7712	MACROD2	Chr20:14070869	1.00	A
cnv_1129	20p13	1556551	1593900	37349	SIRPB1	Chr20:1723766	1.00	T
cnv_1128	20p13	365851	379800	13949	TRIB3	Chr20:116028	1.00	A
cnv_1152	20q13.2	52646851	52658100	11249	BCAS1	Chr20:52629878	1.00	A
cnv_1158	21p11.2	9935101	9963000	27899	TEKT4P2	Chr21:9967732	1.00	C
cnv_91	2p13.3	71342543	71347950	5407	MCEE	Chr2:71332303	1.00	A
cnv_88	2p16.3	48851101	48857952	6851	GTF2A1L, STON1-GTF2A1L	Chr2:48861343	1.00	T
cnv_80	2p25.1	11395188	11400419	5231	ROCK2	Chr2:11321667	1.00	C
cnv_77	2p25.3	3732751	3738025	5274	ALLC	Chr2:3727297	1.00	C
cnv_75	2p25.3	1216801	1227150	10349	SNTG2	Chr2:800643	1.00	C
cnv_76	2p25.3	1527941	1541250	13309	TPO	Chr2:1219281	1.00	G
cnv_118	2q13	114249151	114262200	13049	CBWD2, FOXD4L1	Chr2:114173776	1.00	G
cnv_119	2q14.1	115534801	115542450	7649	DPP10	Chr2:115635069	1.00	C
cnv_147	2q33.1,q33.2	203295601	203312700	17099	BMPR2	Chr2:202988258	1.00	T
cnv_148	2q33.2	203899051	203904450	5399	NBEAL1	Chr2:203706433	1.00	T
cnv_158	2q37.3	242721395	242729375	7980	GAL3ST2	Chr2:242949245	1.00	T
cnv_189	3p11.1	89509964	89516006	6042	EPHA3	Chr3:89922282	1.00	A
cnv_176	3p21.1	53027101	53038800	11699	SFMBT1	Chr3:52971680	1.00	A
cnv_172	3p22.2	37978201	37987200	8999	CTDSPL	Chr3:37962472	1.00	C
cnv_214	3q26.31	175078801	175084200	5399	NAALADL2	Chr3:175067438	1.00	T
cnv_216	3q28	189363151	189370899	7748	TP63	Chr3:189358862	1.00	G
cnv_219	3q29	192304801	192312900	8099	FGF12	Chr3:192295856	1.00	A
cnv_240	4p16.1	9496391	9558683	62292	MIR548I2	Chr4:9241390	1.00	G
cnv_237	4p16.1	9158401	9274500	116099	USP17L15, USP17L11, USP17L20, USP17L19, USP17L18, USP17L12,	Chr4:9364630	1.00	A

Supplemental Table 4. Genotype at 197 WGS Genomic SNPs with r^2>0.75 Tagging a Genic Deletion CNV[1] (cont., page 3)

CNV-ID	ChrCytoband	Start	End	Size	Gene(s)	TAG-Location	MAX r^2	TAG GT
					USP17L10, USP17L17, USP17L21, USP17L13, USP17L22			
cnv_260	4q13.2	69669001	69709050	40049	UGT2B10	Chr4:69545429	1.00	T
cnv_266	4q21.1	78491701	78497550	5849	CXCL13	Chr4:78473616	1.00	T
cnv_275	4q24	107056351	107063550	7199	TBCK	Chr4:106960346	1.00	A
cnv_294	4q32.3	168109201	168115500	6299	SPOCK3	Chr4:168081889	1.00	T
cnv_308	4q35.2	190982701	190995750	13049	DUX4, DUX2, DUX4L2, DUX4L6, DUX4L5, DUX4L3, DUX4L4	Chr4:190968418	1.00	C
cnv_340	5q13.2	68953051	68969250	16199	GUSBP3	Chr5:69371799	1.00	G
cnv_347	5q13.2	69801751	69818400	16649	SMA5, GUSBP9	Chr5:69700788	1.00	T
cnv_362	5q31.3	140221801	140238900	17099	PCDHA1, PCDHA8, PCDHA7, PCDHA10, PCDHA4, PCDHA5, PCDHA9, PCDHA3, PCDHA2, PCDHA6	Chr5:140221098	1.00	G
cnv_372	5q35.3	180375301	180430650	55349	BTNL8, BTNL3	Chr5:180430797	1.00	C
cnv_369	5q35.3	177190201	177200100	9899	FAM153A	Chr5:177339390	1.00	T
cnv_371	5q35.3	178348051	178353450	5399	ZFP2	Chr5:178365247	1.00	C
cnv_396	6p21.32	32522401	32548050	25649	HLA-DRB6, HLA-DRB1	Chr6:32463093	1.00	G
cnv_379	6p22.3	22167451	22176000	8549	LINC00340	Chr6:22157291	1.00	T
cnv_454	7p12.3	48581101	48592800	11699	ABCA13	Chr7:48530497	1.00	A
cnv_498	7q11.23	74300851	74318850	17999	PMS2P5, STAG3L2	Chr7:74346484	1.00	G
cnv_508	7q22.1	100327501	100337850	10349	ZAN	Chr7:100326744	1.00	A
cnv_535	7q36.3	156386701	156394350	7649	LOC100506380	Chr7:156377775	1.00	T
cnv_568	8p11.22	39231901	39387150	155249	ADAM5, ADAM3A	Chr8:39211475	1.00	A
cnv_545	8p23.1	6833251	6871950	38699	DEFA1, DEFA1B, DEFT1P, DEFT1P2	Chr8:7363817	1.00	C
cnv_548	8p23.1	7251751	7355250	103499	DEFB106B, DEFB106A, SPAG11B, DEFB4B, DEFB103A, DEFB104B, DEFB104A, DEFB103B, DEFB107B, DEFB105B, DEFB105A, DEFB107A	Chr8:6838521	1.00	C
cnv_549	8p23.1	7364251	7474050	109799	DEFB107B, FAM90A7P, DEFB107A	Chr8:7072226	1.00	T
cnv_551	8p23.1	7566751	7824150	257399	FAM66E, DEFB107A, DEFB106B, SPAG11B, SPAG11A, DEFB105B, DEFB104B, FAM90A10P,	Chr8:7114448	1.00	C

Supplemental Table 4. Genotype at 197 WGS Genomic SNPs with r^2>0.75 Tagging a Genic Deletion CNV[1] (cont., page 4)

CNV-ID	ChrCytoband	Start	End	Size	Gene(s)	TAG-Location	MAX r^2	TAG GT
					DEFB107B, DEFB106A, DEFB105A, DEFB104A, ZNF705B, DEFB4A, DEFB103A, DEFB103B			
cnv_558	8p23.1	12046501	12091950	45449	FAM86B1	Chr8:11956169	1.00	T
cnv_554	8p23.1	8052751	8087850	35099	FAM86B3P	Chr8:7571541	1.00	A
cnv_547	8p23.1	7111351	7155900	44549	LINC00965	Chr8:7070713	1.00	G
cnv_560	8p23.1	12466351	12504600	38249	LOC729732	Chr8:12353763	1.00	A
cnv_553	8p23.1	7936651	7986600	49949	MIR548I3	Chr8:7440046	1.00	C
cnv_577	8q11.21	51030901	51038550	7649	SNTG1	Chr8:51029371	1.00	G
cnv_582	8q13.3	73787765	73793823	6058	KCNB2	Chr8:73292434	1.00	T
cnv_585	8q21.2	85260966	85269169	8203	RALYL	Chr8:85259662	1.00	T
cnv_587	8q21.3	88267762	88272956	5194	CNBD1	Chr8:87974728	1.00	T
cnv_628	9p11.2	43669624	44180216	510592	CNTNAP3B	Chr9:43378850	1.00	T
cnv_625	9p12	42364351	42374700	10349	ANKRD20A3, ANKRD20A2	Chr9:42141858	1.00	G
cnv_622	9p12	41969701	41984100	14399	KGFLP2	Chr9:41891031	1.00	T
cnv_627	9p12,p11.2	43578451	43852500	274049	CNTNAP3B, SPATA31A6	Chr9:43081246	1.00	A
cnv_610	9p21.1	29092051	29098014	5963	LINGO2	Chr9:29098065	1.00	A
cnv_605	9p22.2	17401051	17406983	5932	CNTLN	Chr9:17297366	1.00	C
cnv_600	9p24.3	590401	608850	18449	KANK1	Chr9:550980	1.00	G
cnv_638	9q12	65489401	65657700	168299	SPATA31A5, SPATA31A7, FAM74A4, FAM74A2	Chr9:66051351	1.00	G
cnv_499	7q11.23	76144951	76165200	20249	UPK3B	Chr7:76171197	0.98	T
cnv_262	4q13.2	70467301	70472700	5399	UGT2A1, UGT2A2	Chr4:70466593	0.98	T
cnv_914	14q13.2	35605351	35615250	9899	KIAA0391	Chr14:35654467	0.97	G
cnv_261	4q13.2	70123501	70232400	108899	UGT2B28	Chr4:70119629	0.97	G
cnv_272	4q22.2	93855151	93861450	6299	GRID2	Chr4:93861928	0.97	C
cnv_204	3q22.1	131708251	131713650	5399	CPNE4	Chr3:131715254	0.97	A
cnv_776	11q11	55364851	55431450	66599	OR4C11, OR4P4, OR4S2	Chr11:55227095	0.96	G
cnv_115	2q11.2	98135551	98162100	26549	ANKRD36B	Chr2:98223415	0.96	C
cnv_135	2q31.1	170105115	170111162	6047	LRP2	Chr2:170099111	0.95	C
cnv_282	4q27	122282101	122290200	8099	QRFPR	Chr4:122270034	0.95	C
cnv_722	10q24.2	100687951	100701900	13949	HPSE2	Chr10:100668400	0.95	A
cnv_775	11q11	55031401	55038600	7199	TRIM48	Chr11:54825337	0.95	T
cnv_961	15q23	71021839	71027508	5669	UACA	Chr15:71048015	0.95	A
cnv_416	6q13	72863551	72873540	9989	RIMS1	Chr6:72827471	0.95	A
cnv_414	6q13	70720219	70726294	6075	COL19A1	Chr6:70715370	0.94	G
cnv_929	14q32.33	106531651	106785900	254249	LINC00226	Chr14:106531261	0.94	G
cnv_217	3q28	191064601	191071800	7199	CCDC50	Chr3:191075656	0.93	A
cnv_199	3q13.31	114656851	114668550	11699	ZBTB20	Chr3:114655252	0.92	T
cnv_14	1p36.11	25589251	25661700	72449	RHD	Chr1:25561667	0.92	A
cnv_412	6q12	66398851	66404700	5849	EYS	Chr6:66348218	0.92	C
cnv_897	13q33.3	108946801	108951810	5009	TNFSF13B	Chr13:108937313	0.92	C
cnv_1113	19q13.32	46622701	46628312	5611	IGFL3	Chr19:46618665	0.90	A
cnv_134	2q24.3	167844952	167851208	6256	XIRP2	Chr2:167832996	0.90	T
cnv_561	8p22	15418801	15435450	16649	TUSC3	Chr8:15418318	0.90	G
cnv_141	2q31.2	180411301	180422100	10799	ZNF385B	Chr2:180399774	0.90	T
cnv_50	1q21.3	152555401	152587800	32399	LCE3C, LCE3B	Chr1:152589499	0.89	G

Supplemental Table 4. Genotype at 197 WGS Genomic SNPs with r^2>0.75 Tagging a Genic Deletion CNV[1] (cont., page 5)

CNV-ID	ChrCytoband	Start	End	Size	Gene(s)	TAG-Location	MAX r^2	TAG GT
cnv_1186	22q11.23	24314401	24334650	20249	GSTT2, DDTL, DDT	Chr22:24294903	0.89	G
cnv_824	12p11.23	27648001	27655200	7199	SMCO2	Chr12:27647382	0.89	T
cnv_43	1q21.1	144461251	144484200	22949	LOC100288142, LOC728875	Chr1:144512411	0.89	G
cnv_151	2q34	213183901	213192011	8110	ERBB4	Chr2:213143798	0.89	C
cnv_361	5q31.3	140096701	140103900	7199	VTRNA1-2	Chr5:140154668	0.89	C
cnv_32	1p13.2	114039827	114045877	6050	MAGI3	Chr1:113974263	0.89	G
cnv_375	6p25.3	256501	296100	39599	DUSP22	Chr6:126196	0.89	T
cnv_206	3q22.3	136021013	136026198	5185	PCCB	Chr3:136053963	0.88	G
cnv_919	14q21.3	50717701	50727150	9449	L2HGDH	Chr14:50529363	0.87	T
cnv_290	4q31.3	151875451	151885800	10349	LRBA	Chr4:151750966	0.87	C
cnv_1023	16q23.3	83670839	83676912	6073	CDH13	Chr16:83677291	0.86	G
cnv_432	6q23.2	132707251	132712650	5399	MOXD1	Chr6:132698520	0.85	C
cnv_1073	18q11.2	23746051	23751450	5399	PSMA8	Chr18:23739314	0.85	C
cnv_247	4p15.2	21368701	21377250	8549	KCNIP4	Chr4:21344645	0.85	T
cnv_359	5q31.1	132918964	132924990	6026	FSTL4	Chr5:132925285	0.85	A
cnv_1021	16q23.1	78371551	78384600	13049	WWOX	Chr16:78384896	0.82	C
cnv_730	10q26.3	133098751	133106400	7649	TCERG1L	Chr10:133051977	0.82	A
cnv_1163	21q11.2	14447251	14557050	109799	ANKRD30BP2	Chr21:14366491	0.82	C
cnv_591	8q22.3	102620701	102627000	6299	GRHL2	Chr8:102617205	0.82	T
cnv_698	10q11.22	47938051	47951100	13049	FAM21B	Chr10:48192575	0.82	A
cnv_203	3q22.1	130347566	130353108	5542	COL6A6	Chr3:130333400	0.82	A
cnv_497	7q11.23	72270262	72275850	5588	TYW1B	Chr7:72228291	0.81	C
cnv_671	10p12.33	17955901	17974350	18449	MRC1	Chr10:18222481	0.81	T
cnv_140	2q31.2	180066601	180081450	14849	SESTD1	Chr2:180065665	0.80	A
cnv_990	16p12.3	18832524	18838376	5852	SMG1	Chr16:18938730	0.80	A
cnv_793	11q14.3	89809651	89827650	17999	UBTFL1	Chr11:89781495	0.80	A
cnv_385	6p22.1	29851201	29911240	60039	HLA-H, HLA-A, HCG4B	Chr6:29848186	0.79	G
cnv_860	12q24.12	112012651	112027500	14849	ATXN2	Chr12:111861436	0.79	G
cnv_895	13q31.3	93009151	93019050	9899	GPC5	Chr13:92965986	0.79	G
cnv_354	5q22.1	110903851	110913750	9899	STARD4-AS1	Chr5:110903520	0.79	A
cnv_402	6p12.1	53855101	53866350	11249	MLIP-IT1	Chr6:54156708	0.79	G
cnv_383	6p22.1	29091151	29161800	70649	OR2J2	Chr6:28942785	0.79	A
cnv_581	8q13.2	68156551	68161950	5399	ARFGEF1	Chr8:67841707	0.79	A
cnv_667	9q34.3	140976001	140982300	6299	CACNA1B	Chr9:140983283	0.79	T
cnv_652	9q21.11	71738101	71743500	5399	TJP2	Chr9:71733650	0.79	A
cnv_963	15q23	72379351	72400950	21599	MYO9A	Chr15:72332917	0.79	G
cnv_259	4q13.2	69373801	69490800	116999	UGT2B17	Chr4:69491183	0.78	T
cnv_1103	19q13.12	35849701	35863650	13949	FFAR3	Chr19:35870780	0.78	T
cnv_431	6q22.33	129319515	129325582	6067	LAMA2	Chr6:129330852	0.78	A
cnv_695	10q11.22	47241451	47281050	39599	BMS1P2, BMS1P6	Chr10:47207415	0.78	C
cnv_696	10q11.22	47408401	47415600	7199	FAM35DP	Chr10:47207415	0.78	C
cnv_954	15q13.3	32865751	32885550	19799	LOC100996255	Chr15:32689004	0.77	C
cnv_1	1p36.33	40051	114300	74249	OR4F5	Chr1:533869	0.77	A
cnv_536	7q36.3	158123701	158134551	10850	PTPRN2	Chr7:158631645	0.77	G
cnv_72	1q44	248725801	248798250	72449	OR2T10, OR2T34, OR2T11	Chr1:248848132	0.75	A
cnv_815	12p13.31	8539485	8549586	10101	LINC00937	Chr12:8561818	0.75	G

[1] CNV-ID, unique ID given to 1193 common deletions; ChrCytoband: chromosome and cytoband; Start, End: Start and end coordinate of the CNV; Size: End minus Start; Gene(s): RefSeq gene(s) affected by CNVs; TAG-Location: chromosome and position coordinate of nearest tagging SNP; MAX r^2: Maximum pairwise correlation of the tagging SNP to the CNV; TAG-GT: The genotype at the tagging SNP position which correlates with the deletion allele.

Supplemental References

1. Klambauer G, Schwarzbauer K, Mayr A, Clevert DA, Mitterecker A, Bodenhofer U, Hochreiter S. cn.MOPS: mixture of Poissons for discovering copy number variations in next-generation sequencing data with a low false discovery rate. Nucleic Acids Res. 2012,40:e69.

2. Kanehisa M, Goto S, Sato Y, Furumichi M, Tanabe M. KEGG for integration and interpretation of large-scale molecular data sets. Nucleic Acids Res. 2012,40:D109-D114.

Supplemental Figure Legends

Supplemental Figure S1. Distribution of “raw” CNVs detected in the initial cohort of 100 individual as described in Methods. This quality control step was imposed to detect and remove outlier samples, which could skew the dataset. In this cohort of 100 Qataris, 3 Q1’s had >10,000 CNVs called per genome, affecting up to 730Mb of the genome, and were removed from the cohort prior to CNV processing and population-level analysis.

Supplemental Figure S2. Number of CNVs called by each platform. **A.** Contribution of each of the four different algorithms to the final CNV content detected in a median individual. Venn diagram illustrates the overlap between the four platforms used to detect CNVs in this cohort. Whole genome sequencing CNVs were called using cn.MOPS and calls provided by the Illumina Genome Network sequencing service; 2.5M Omni Array CNVs were called using QuantiSNP and Illumina’s proprietary GenomeStudio software package – cnvPartition. CNVs are considered to overlap if at least 50% of their lengths overlap. **B.** Overlap of CNVs detected by each algorithm in generating population-level CNVRs. Venn Diagram illustrates the size of overlap between the four platforms - whole genome sequencing platforms on the left and Array platforms on the right - based on population merged CNVs (Final individual CNVs in each subpopulation are merged at 50%, and the resulting CNVRs from all 3 sub-populations are used to produce the platform overlap counts).

Supplemental Figure S3. Principal component analysis based on CNV sharing in all 97 individuals. 57 Q1 subjects appear in black, 20 Q2 in red, and 20 Q3 in green.

Supplemental Figure S4. Distribution of ‘final’ CNVs per individual in 97 Qataris. See methods for detailed QC and strategy to generate these individual CNV files prior to population merge. CN frequencies appear by class in all individuals in all 3 sub-populations Q1 (n=57), Q2

(n=20) and Q3 (n=20). Bars are color-coded by CN Class, Blue = 0, Red = 1, Green = 3, and Black = 4+. Dashed lines separate the three subpopulations.

Supplemental Figure S5. Kegg Pathways Diagrams obtained from [2] showing key genes in disease-relevant pathways affected by CNVs in Qataris. Five of the top 15 CNV-affected pathways in Qataris may be relevant to public health, these include: **A.** type II diabetes mellitus pathway; **B.** insulin signaling pathway; **C.** starch and sucrose metabolism; **D.** type I diabetes; **E.** Notch pathway. Red stars denote genes within each pathway that are affected by CNVs in Qataris.

Supplemental Figure 1

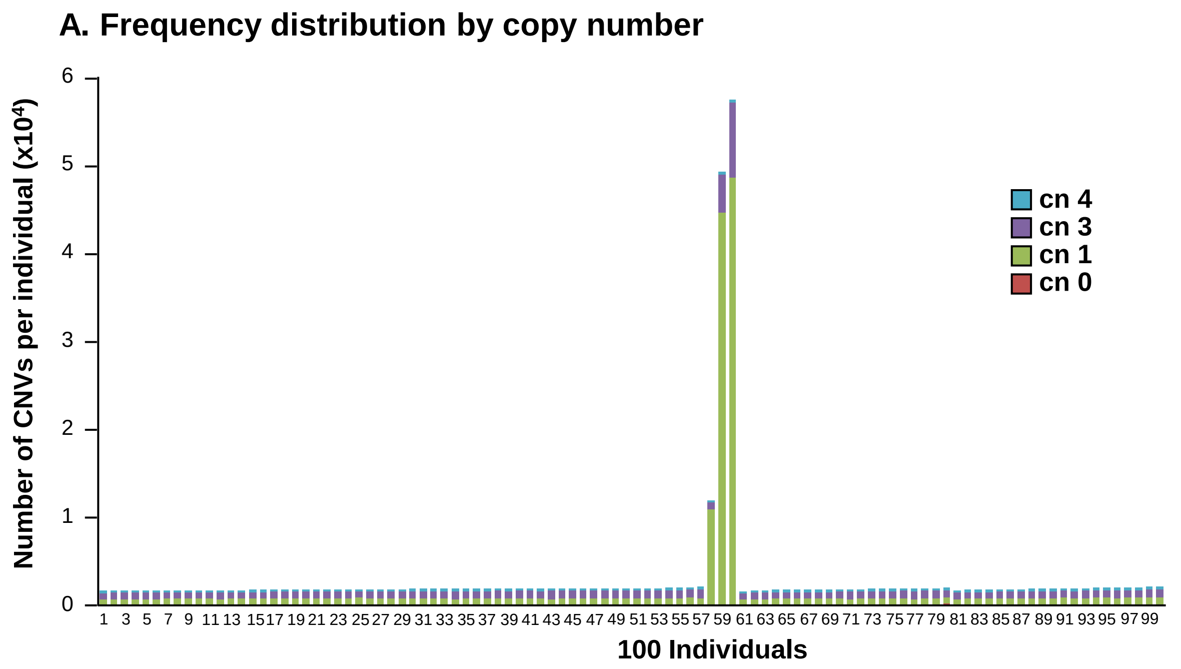

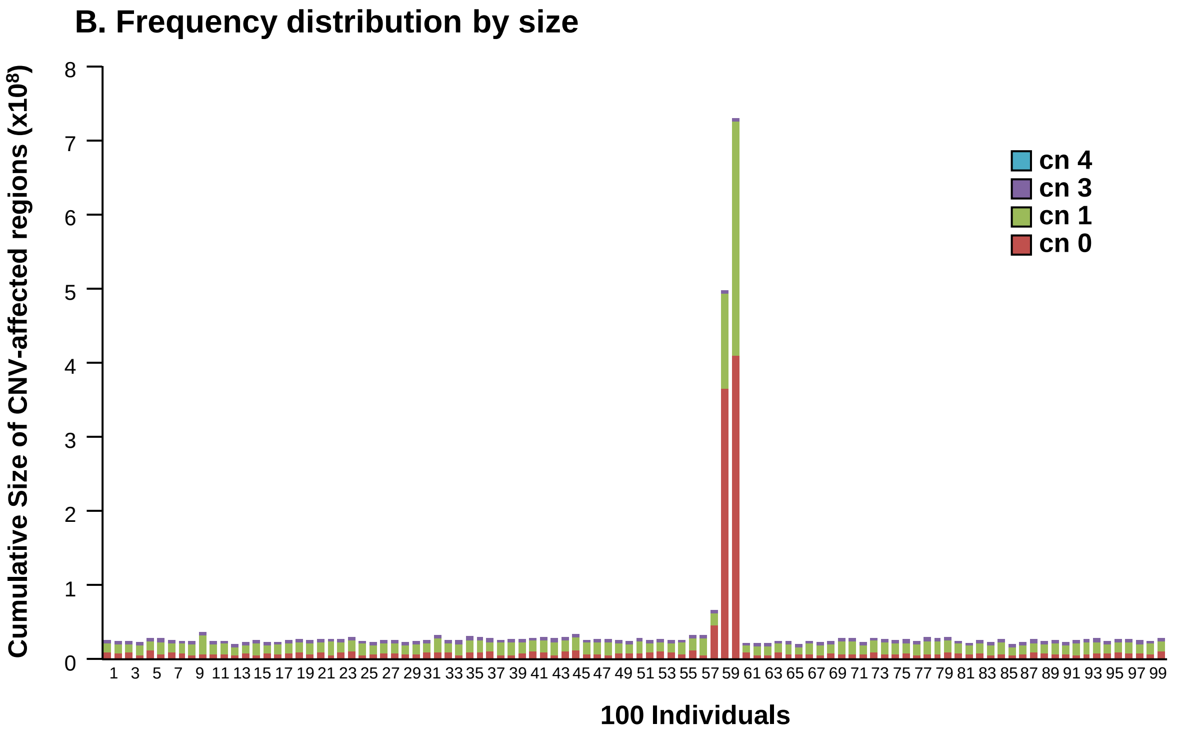

Supplemental Figure 2

A.

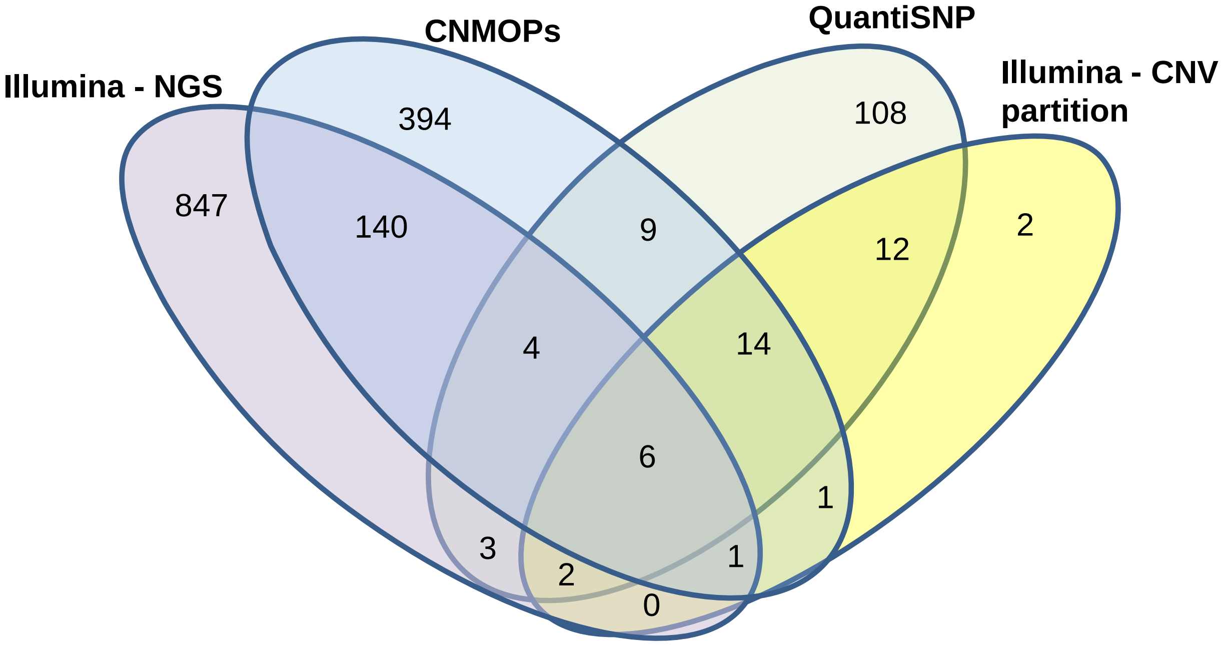

B.

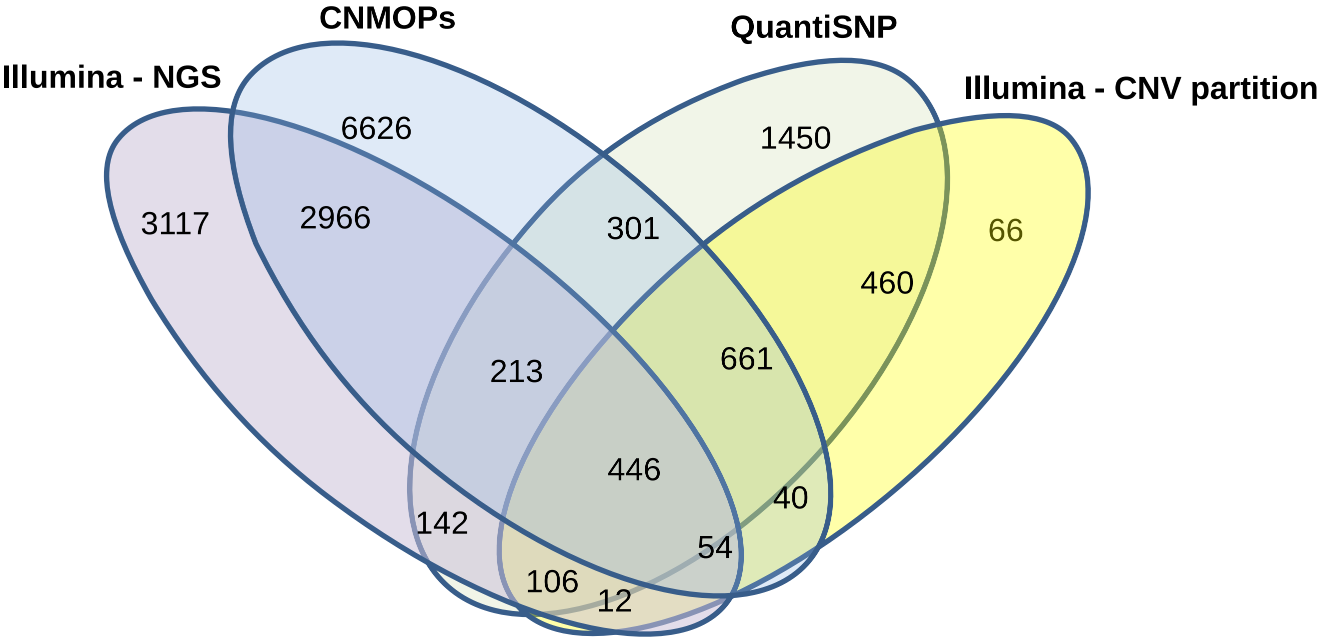

Supplemental Figure 3

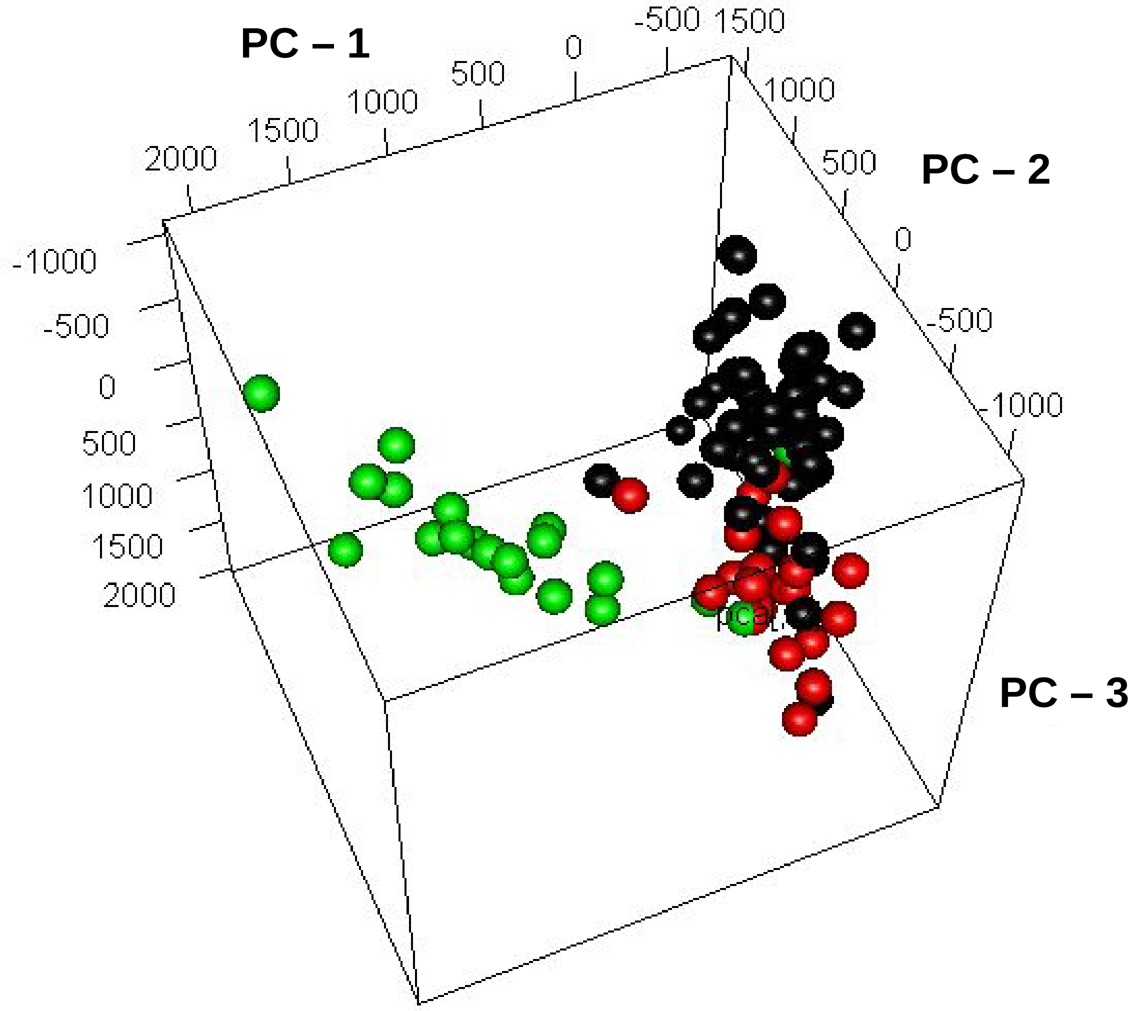

Supplemental Figure 4

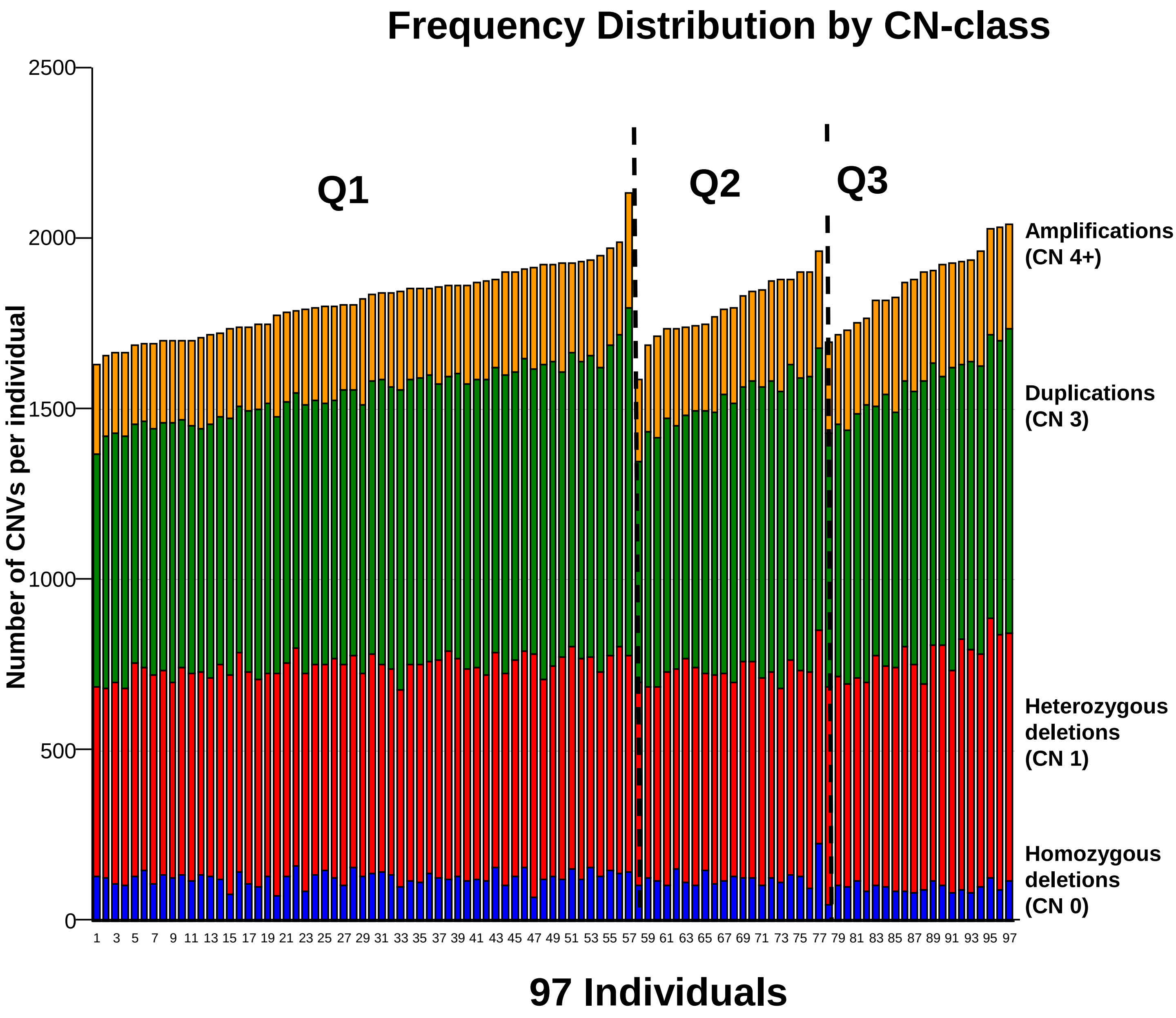

Supplemental Figure 5A, B

A.

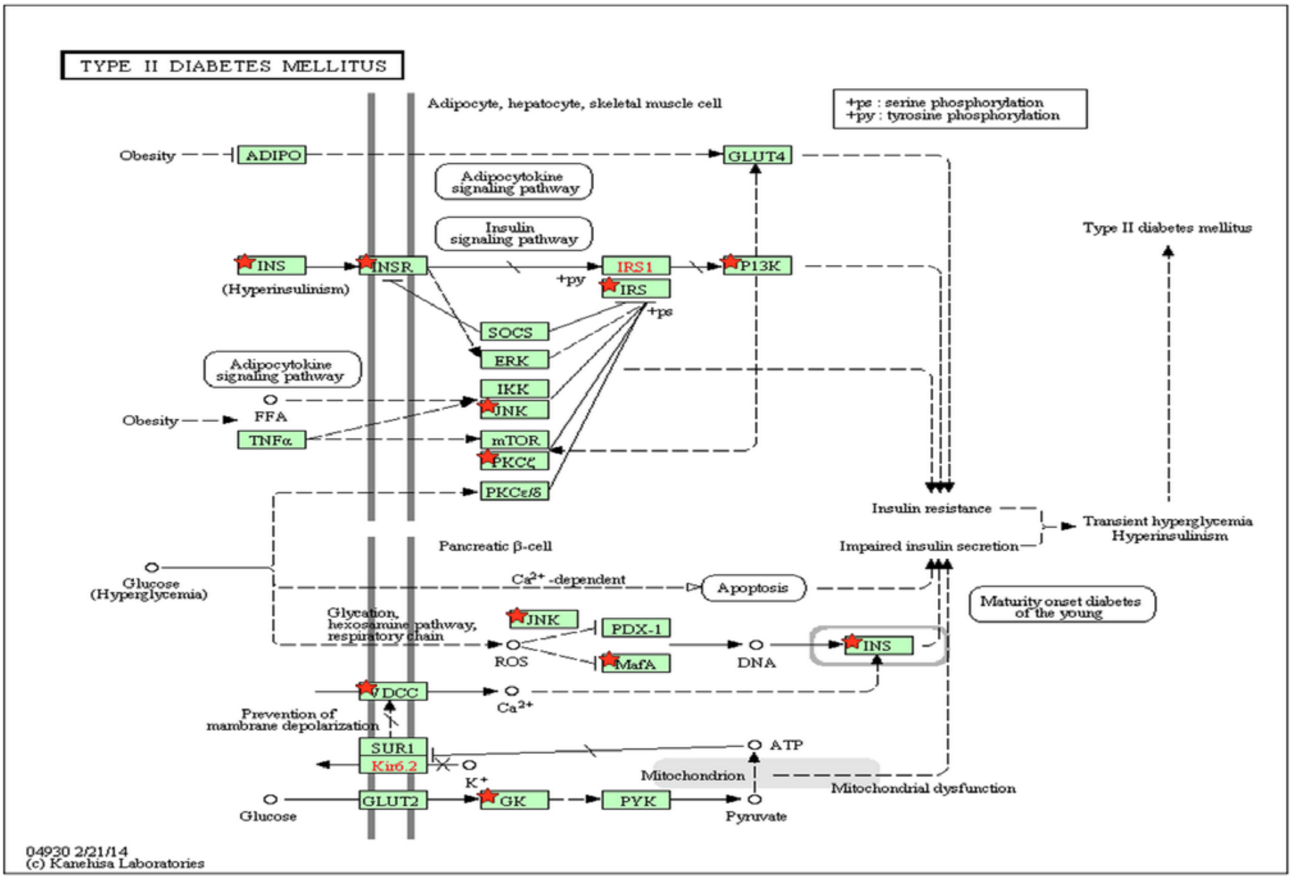

B.

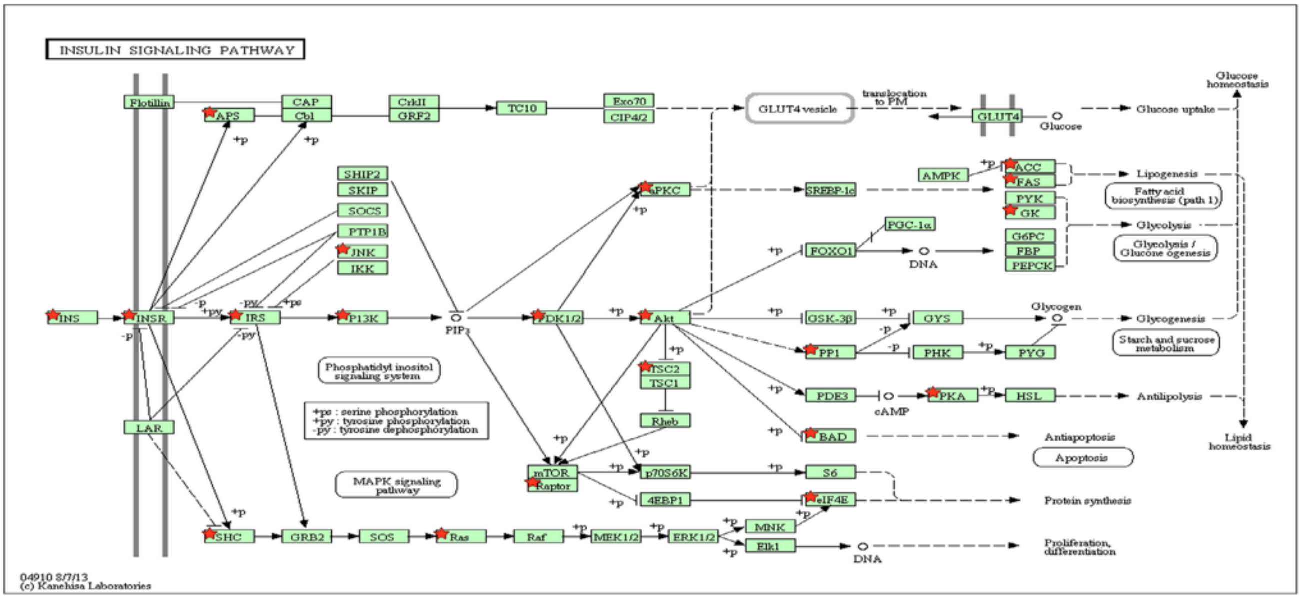

Supplemental Figure 5C, D

C.

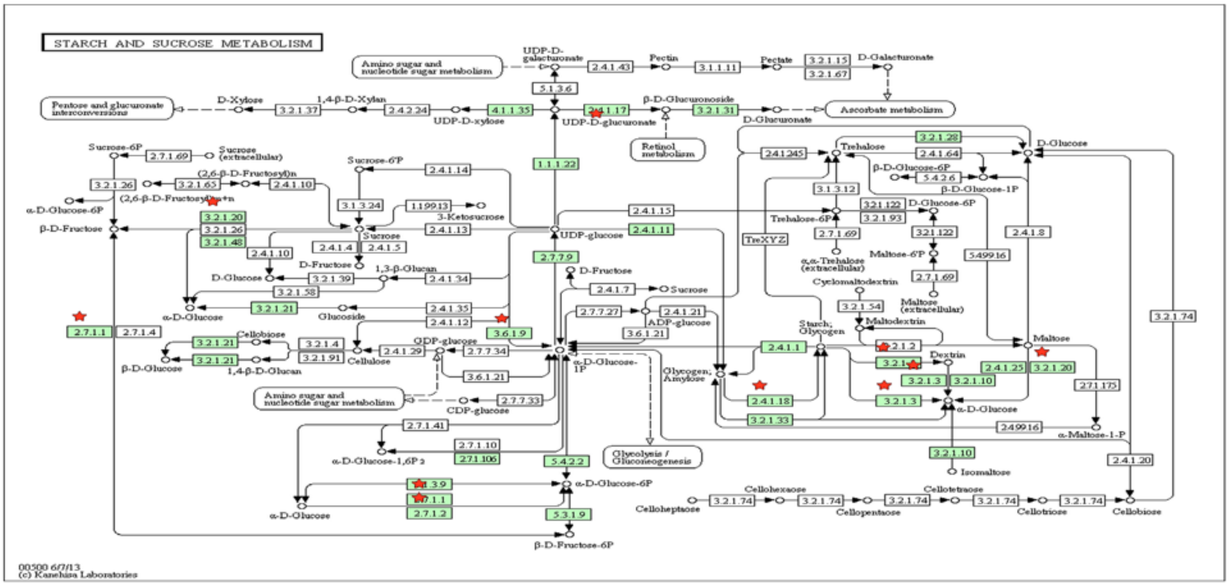

D.

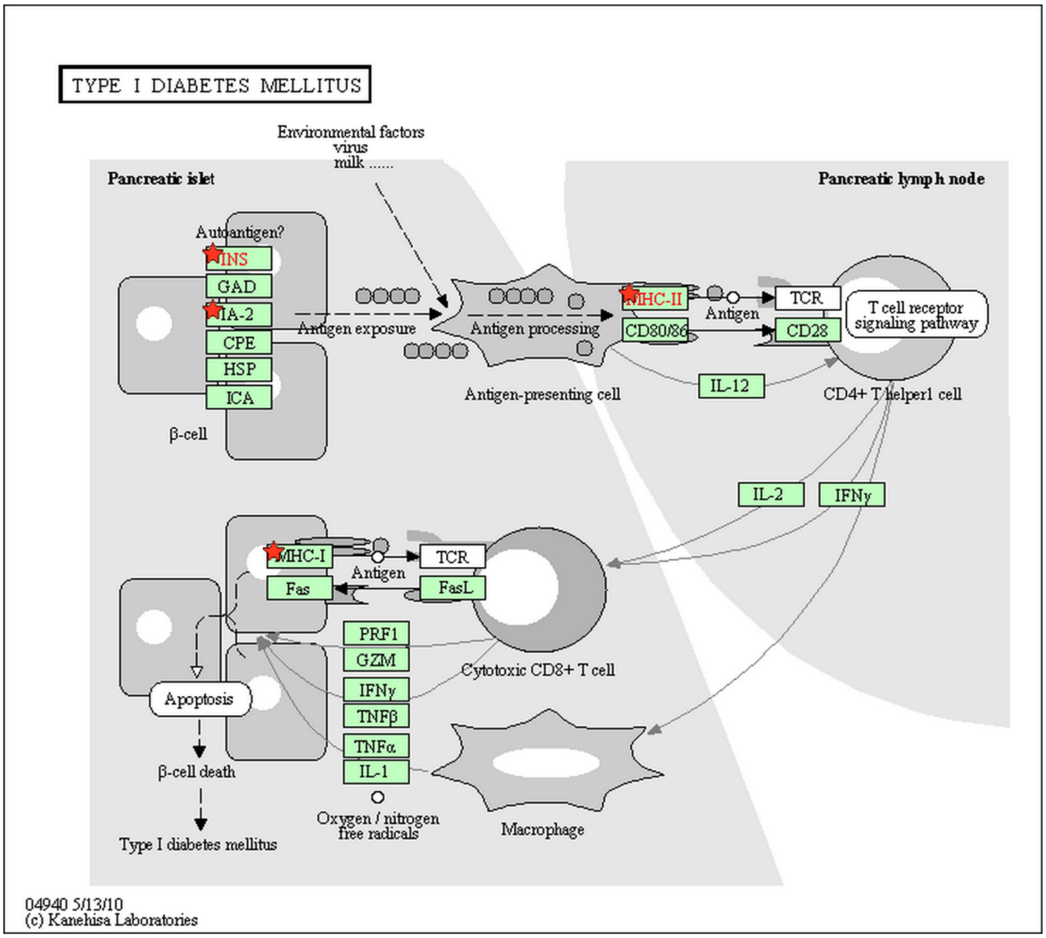

E.

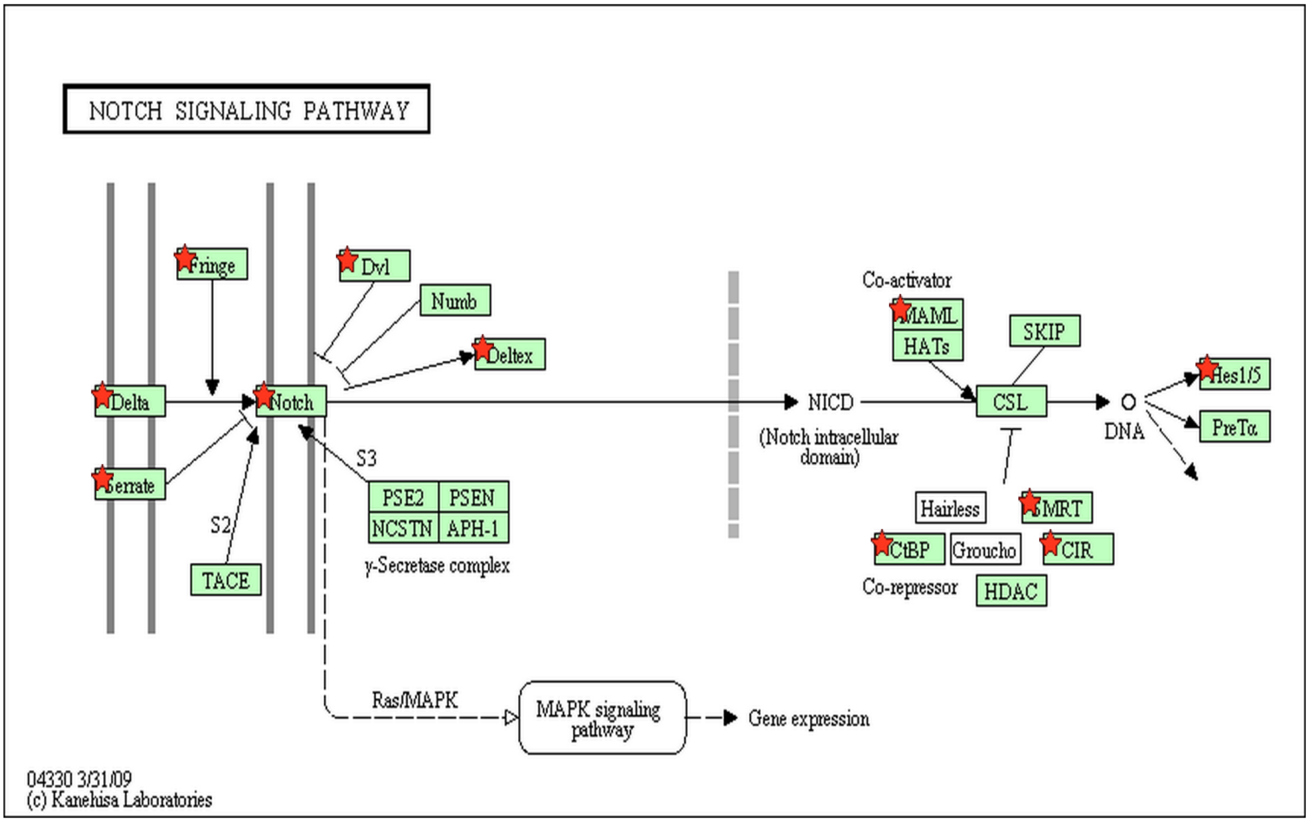
NOTCH SIGNALING PATHWAY
Fringe
Dvl
Numb
Deltex
Delta
Notch
Serrate
S2
TACE
S3
PSE2
PSEN
NCSTN
APH-1
γ-Secretase complex
NICD
(Notch intracellular domain)
Co-activator
MAML
HATs
SKIP
CSL
DNA
Hes1/5
PreTα
Hairless
SMRT
CtBP
Groucho
CIR
Co-repressor
HDAC
Ras/MAPK
MAPK signaling pathway
Gene expression
04330 3/31/09
(c) Kanehisa Laboratories
